# The regioselective synthesis of spirooxindolo pyrrolidines and pyrrolizidines via three-component reactions of acrylamides and aroylacrylic acids with isatins and α-amino acids

**DOI:** 10.3762/bjoc.10.8

**Published:** 2014-01-09

**Authors:** Tatyana L Pavlovskaya, Fedor G Yaremenko, Victoria V Lipson, Svetlana V Shishkina, Oleg V Shishkin, Vladimir I Musatov, Alexander S Karpenko

**Affiliations:** 1 State Scientific Institution “Institute for Single Crystals” of National Academy of Sciences of Ukraine, 60, Lenin ave., Kharkov, 61178, Ukraine; 2 Antidiabetic Drug Laboratory, State Institution “V.J. Danilevsky Institute of Problems of Endocrine Pathology at the Academy of Medical Sciences of Ukraine”, 10, Artem St., Kharkov, 61002, Ukraine; 3 Organic Chemistry Department, V.N. Karazin Kharkov National University, 4, Svobody Sq., 61077, Kharkov, Ukraine,; 4 A.V. Bogatsky physico-chemical institute of the National Academy of Sciences of Ukraine, 86, Lustdorfskaya doroga, 65080, Odessa, Ukraine

**Keywords:** acrylamides, aroylacrylic acids, azomethine ylide, cyclative rearrangement, cycloaddition, multicomponent, spirooxindoles

## Abstract

The regioselective three-component condensation of azomethine ylides derived from isatins and α-amino acids with acrylamides or aroylacrylic acids as dipolarophiles has been realized through a one-pot 1,3-dipolar cycloaddition protocol. Decarboxylation of 2'-aroyl-2-oxo-1,1',2,2',5',6',7',7a'-octahydrospiro[indole-3,3'-pyrrolizine]-1'-carboxylic acids is accompanied by cyclative rearrangement with formation of dihydropyrrolizinyl indolones.

## Introduction

The design of new spirocyclic compounds is intriguing due to their unique non-planar structure and great potential for binding to biomolecules due to their inherent rigid chiral structure. The spirooxindolo pyrrolidine and pyrrolizidine frameworks form core units of many naturally occurring molecules possess significant pharmacological activities. Among them are alkaloids such as horsfiline from *Horsfieldia superbа* [1–3], elacomine from *Elaeagnus commutatа* [4], mitraphylline from *Uncaria tomentosa* [5] and spirotryprostatines A and B from the secondary metabolites of *Aspergillus fumigatus* [6–8]. In particular, oxindole derivatives are well known as powerful anti-tumor agents due to their kinase inhibitory properties, especially as tyrosine kinase inhibitors [9–10]. The multicomponent 1,3-dipolar cycloaddition of azomethine ylides, generated in situ via decarboxylative condensation of isatins and α-amino acids with olefinic and acetylenic dipolarophiles, represents a key approach for the regio- and stereoselective construction of a variety of complex spirooxindoles. Recently, this route has become significant in combinatorial chemistry due to its process simplicity, mild conditions, atomic economy and extension of the scope of substrates. A large number of focused libraries of spirooxindolo pyrrolidines and pyrrolizidines containing a wide set of natural and nonnatural α-amino acids [11–13], more than fifteen isatins [14], and 1,3-dipolarophiles, e.g. α,β-unsaturated ketones [15–17], maleimides [18–19], benzo[*b*]thiophene-1,1-dioxide [20], bis(arylmethylidene)acetones and -cycloalkanones [21–22], 1,4-naphthoquinone [23], arylidenemalonodinitriles [24], arylidenerhodanines [25–26], α,β-unsaturated lactones [27], nitrostyrenes [28], acrylic and propiolic esters [29], acrylonitriles [30] and arylidene-1,3-dimethylpyrimidine-2,4,6-triones [31] have been reported. However, the molecular diversity of suitable building blocks for construction of spirooxindoles is by far not exhausted with the above mentioned substances. Our interest to spirooxindoles is inspired by the search of new antidiabetic substances that might inhibit 11β-hydroxysteroid dehydrogenase type I (11β-HSD1) in metabolically relevant tissues such as liver and adipose tissue. Recent studies have demonstrated that 11β-HSD1 is a novel molecular target for treating the “metabolic syndrome” and type 2 diabetes mellitus, and that compounds inhibiting the activity of this enzyme provide promising opportunities for the development of therapeutic interventions [32–33]. Among the large class of 11β-HSD1 inhibitors there are compounds containing a pyrrolidine-2-one as a part of the spirocyclic system [34].

In the present work we report the synthesis of spirooxindolo pyrrolidines and pyrrolizidines by utilizing a 1,3-dipolar cycloaddition of hitherto uninvestigated acrylamides and aroylacrylic acids with azomethine ylides, generated in situ via decarboxylative condensation of isatins and N-substituted α-amino acids (sarcosine, proline and thiazolidine-4-carboxilic acid) in a three-component fashion.

## Results and Discussion

The three-component condensation of equimolar amounts of isatins **1**, α-amino acids **2** and acrylamides **3** in boiling aqueous methanol (1:3) afforded the spirooxindoles **4a–4g** in moderate to excellent yields (Table 1). The reaction times largely depend on the reactivity of the employed α-amino acid. The longest reaction time (7 h) was found for sarcosine, while the fastest reaction (40 min) was found for proline as a substrate (Table 1, entries 1 and 3).

**Table 1:** Three-component synthesis of spirooxindoles **4a–4g**.

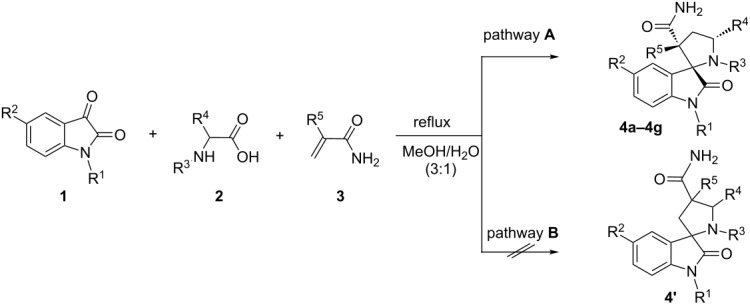								

entry	compound	R^1^	R^2^	R^3^	R^4^	R^5^	yield (%)	time

1	**4a**	H	Br	CH_3_	H	H	31	7 h
2	**4b**	H	NO_2_	CH_2_CH_2_CH_2_		H	85	3 h
3	**4c**	H	NO_2_	CH_2_CH_2_CH_2_		CH_3_	37	40 min
4	**4d**	H	Br	CH_2_SCH_2_		H	42	1 h
5	**4e**	H	NO_2_	CH_2_SCH_2_		H	60	6 h
6	**4f**	4-CH_2_C_6_H_4_Cl	H	CH_2_SCH_2_		H	38	2 h
7	**4g**	CH_3_	Br	CH_2_CH_2_CH_2_		CH_3_	58	2 h

The 1,3-dipolar cycloaddition of unsymmetrical dipolarophiles such as acrylamides can occur via the two pathways **A** and **B** leading to the formation of the regioisomers **4** and **4**’. In our case, spirooxindol **4** is exclusively formed. All new cycloadducts obtained by the above method were characterized by mass spectrometry, ^1^H and ^13^C NMR, and elemental analyses. The regiochemical outcome of the cycloaddition was unambiguously confirmed by NOE experiments in ^1^H NMR as well as later by a single crystal X-ray structure analysis of the cycloadduct **4a**.

**Figure 1:**
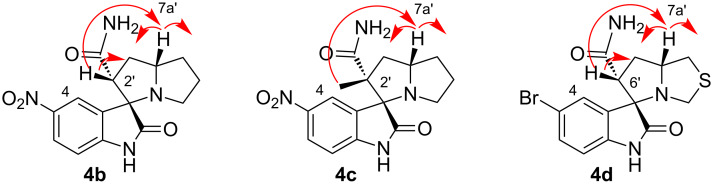
The NOE correlations of the signals in ^1^H NMR spectra of compounds **4b–4d**.

The ^1^H NMR spectra of compounds **4b–4d** have two multiplets at 4.07–3.72 ppm for 7a’-CH and 3.50–3.35 ppm for 2’-CH (compound **4b**) or 6’-CH (compound **4d**) and a singlet at 1.45 ppm for 2’-CCH_3_ of compound **4c**. The relative stereochemistry of compounds **4b–4d** was established by NOE cross peaks between 7a’-CH and 2’(6’-CH) and 2’-CCH_3_. Also, multiplets for 7a’-CH and 2’-(6’-CH) and singlet for 2’-CCH_3_ show correlation signals to the neighboring methylene groups. Additionally, the absence of the NOE cross peak of 4-CH of the isatin nucleus and 2’(6’-CH) or 2’-CCH_3_ of the pyrrolizidine moiety was indicative for the assigned relative stereochemistry. Therefore, the relative stereochemistry could be as shown in Figure 1.

The NH-proton of the oxindole moiety appeared as a singlet between 10.38–10.86 ppm. The ^13^C NMR spectra of compounds **4a–4g** showed characteristic peaks at 71–73 ppm due to the spiro carbon nucleus.

**Figure 2:**
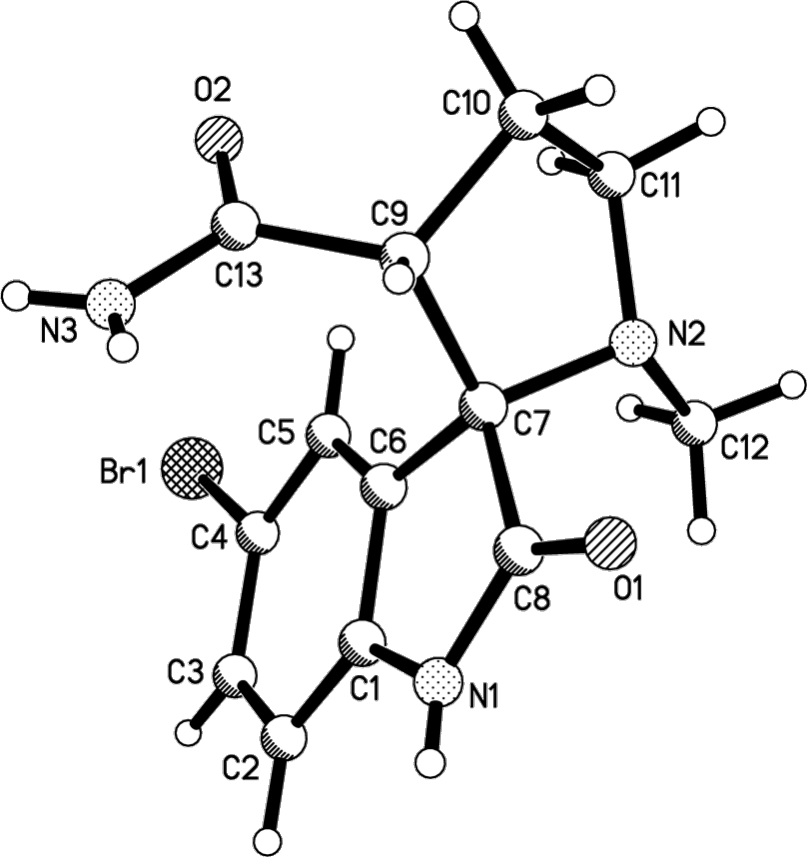
Molecular structure of spirooxindole **4a** according to X-ray diffraction data.

The structure of compound **4a** was determined by an X-ray diffraction study of a single crystal and supports the structure deduced from NMR spectroscopy (Figure 2).

Dipolarophiles, such as aroylacrylic acids **5**, can also be successfully used in this three-component reaction. The cycloaddition of dipolarophiles **5** with non-stabilized azomethine ylides generated from isatins **1** and sarcosine/proline has led to spiropyrrolidines **6a,6b** and spiropyrrolizidines **6c–6h** in moderate to good yields. In this reaction also two regioisomers can be expected, but in all experiments solely the regioisomer **6** is isolated without detectable trace amounts of other isomers.

The higher reactivity of aroylacrylic acids induces remarkable rate acceleration and decreases the reaction time to only 10–15 min in a boiling mixture of methanol and water. The low to moderate yields of the target compounds **6c–6h** can be explained by considerable resinification of the reaction mixture and by formation of byproducts. To suppress these negative adverse processes we carried out the reaction under stirring at room temperature. The results are shown in Table 2.

**Table 2:** Synthesis of spirooxindoles **6a–6h** from the three-component reaction.

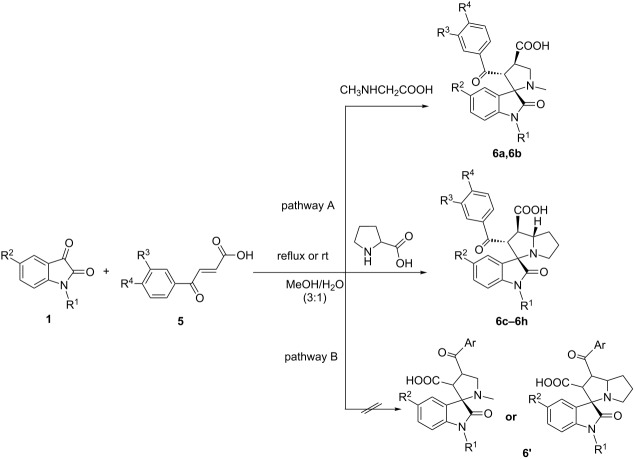									

entry	compound	R^1^	R^2^	R^3^	R^4^	reflux		room temperature (rt)	
						yield (%)	time	yield (%)	time

1	**6a**	H	Br	Cl	Cl	50	6 h	–	–
2	**6b**	CH_3_	Br	H	Br	34	8 h	–	–
3	**6c**	H	H	Cl	Cl	27	30 min	67	12 h
4	**6d**	CH_3_	CH_3_	Cl	Cl	30	1 h	–	–
5	**6e**	H	H	H	Br	15	1 h	57	25 min
6	**6f**	H	H	H	NO_2_	74	10 min	76	3 h
7	**6g**	H	CH_3_	H	NO_2_	30	20 min	74	3 h
8	**6h**	H	Br	H	NO_2_	50	15 min	76	5 h

**Figure 3:**
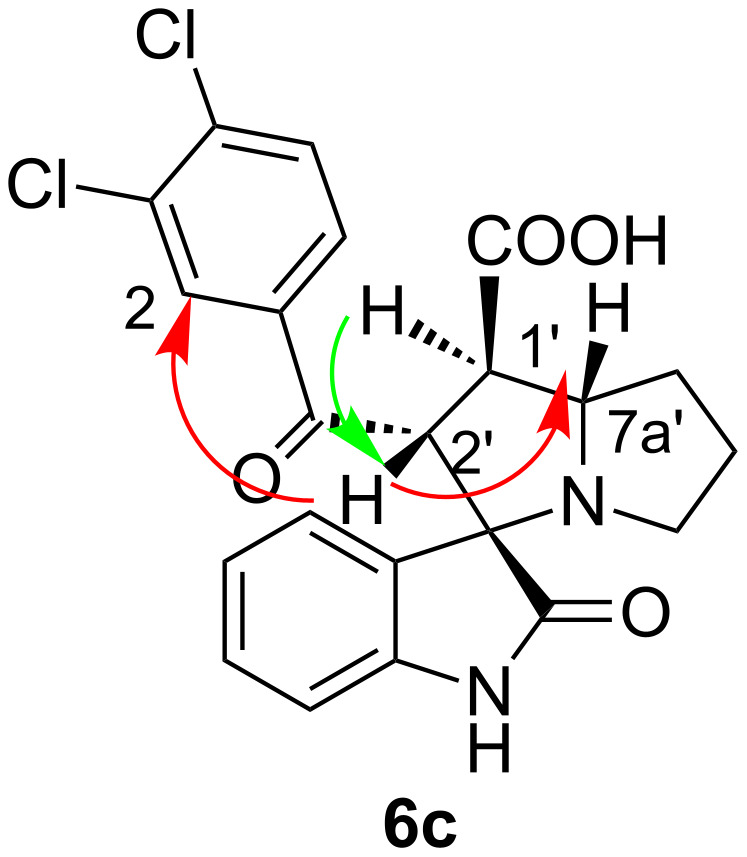
The NOE correlations of the signals in ^1^H NMR spectrum of compound **6c**.

All compound structures are fully supported by spectroscopic data and elemental analysis as illustrated for compound **6c**. The ^1^H NMR spectrum of compound **6c** shows a doublet at 4.66 ppm (*J* =11.4 Hz) for 2’-CH and two multiplets at 3.77–3.93 ppm for 7a’-CH and 3.60–3.43 ppm for 1’-CH. The stereochemistry of compound **6c** was assigned by NOE cross peaks between 7a’-CH and 2’-CH and as well as the neighboring 7’-CH_2_ with a multiplet at 1.91–2.12 ppm. Although a week NOE correlation was found between 1'-CH and 2'-CH, the *trans*-configuration of the mentioned protons is predetermined by the *trans*-configuration of the initial aroyl acrylic acid. Also, a NOE correlation is found between signals of 2’-CH and doublet at 7.30 ppm (*J* =1.8 Hz) for 2-CH of the aroyl acrylic acid moiety. In addition, the absence of NOE cross peaks between 4-CH of the isatin core and 2’-CH of the pyrrolizidine fragment supports the assignment. The NH-proton of the oxindole moiety and the 1’-COOH proton of the pyrrolidine/pyrrolizidine ring give singlets at 10.25 and 12.67 ppm, respectively. Therefore, the correct stereochemistry can be drawn as shown in Figure 3. The ^13^C NMR spectrum of compound **6c** shows a characteristic peak at 73 ppm due to the spiro carbon nucleus.

**Figure 4:**
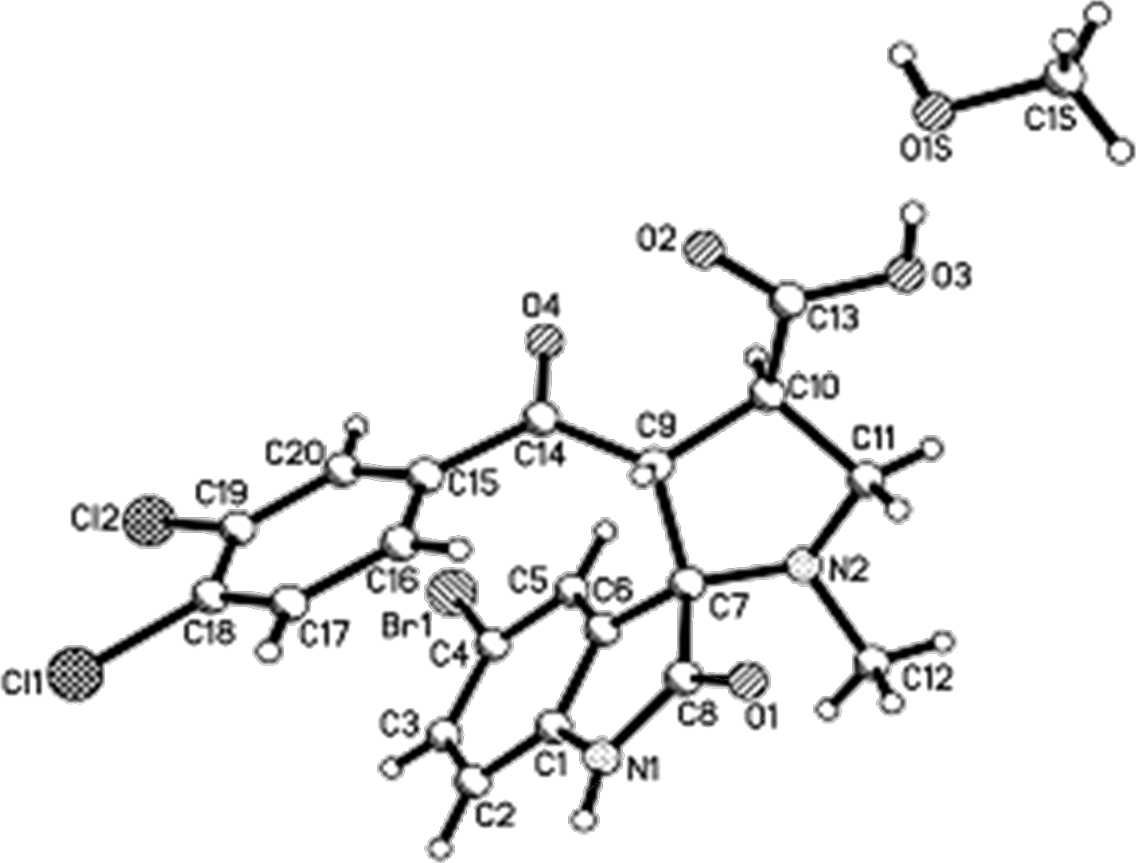
Molecular structure of spirooxindole **6a** observed in crystal phase as solvate with methanol according to X-ray diffraction data.

A single crystal X-ray study of compound **6a** provided a conclusive support for the assigned structure (Figure 4). Interesting feature of this structure is a pincers-like conformation of the molecule. The substituent at the C9 atom has equatorial orientation (the N2–C7–C9–C14 torsion angle is 122.7(2)°) and its carbonyl group is almost coplanar to the C9–C10 endocyclic bond (the C10–C9–C14–O4 torsion angle is 10.2(4)°). Such an orientation of this substituent creates conditions for appearance of intramolecular stacking interactions between the aromatic rings of the indole fragment and the aryl substituent (angle between planes of aromatic rings is 22.9° and the shortest distance between carbon atoms (C6…C15) is 3.04 Å).

**Scheme 1:**
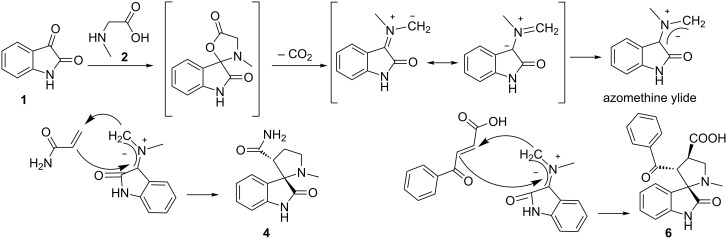
The mechanism of the regioselective synthesis of compounds **4** and **6**.

The mechanism of the azomethine ylide formation by a decarboxylative route has been repeatedly described by a number of authors and is depicted in Scheme 1 [35–36]. The reaction between isatin and the α-amino acid affords the azomethine ylide, which regioselectively adds to the C=C bond of acrylamide or aroylacrylic acid.

**Figure 5:**
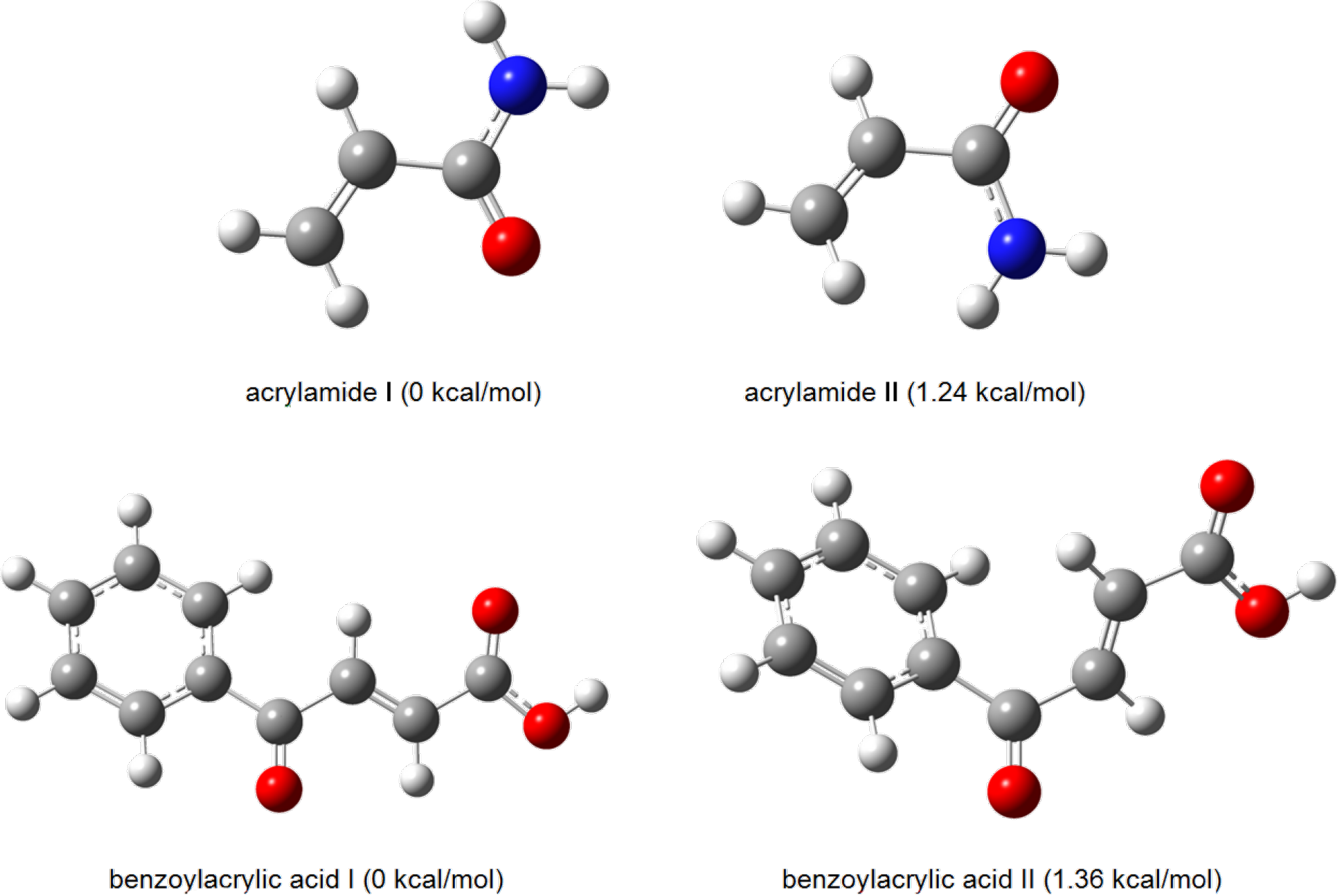
Conformations of acrylamide and benzoylacrylic acid.

Since the stereochemistry of the cycloadducts **4a** and **6a** was clarified by a single-crystal X-ray analysis, the structures of the reacting systems – the azomethine ylide and dipolarophiles (acrylamide and benzoylacrylic acid) – were investigated computationally. The geometrical structures of all possible conformers of the reacting systems were optimized using M06-2X [37] theory with the cc-pVTZ basis set [38] in the GAUSSIAN09 program [39]. The character of stationary points on the potential energy surface was verified by calculations of vibrational frequencies within the harmonic approximation, using analytical second derivatives at the same level of theory. All stationary points possess zero imaginary frequencies. It was found that the acrylamide conformer **I** was more stable than conformer **II** by 1.24 kcal/mol. The most stable conformation of benzoylacrylic acid possesses the benzoyl and carboxylic groups *trans* to each other (Figure 5).

**Figure 6:**
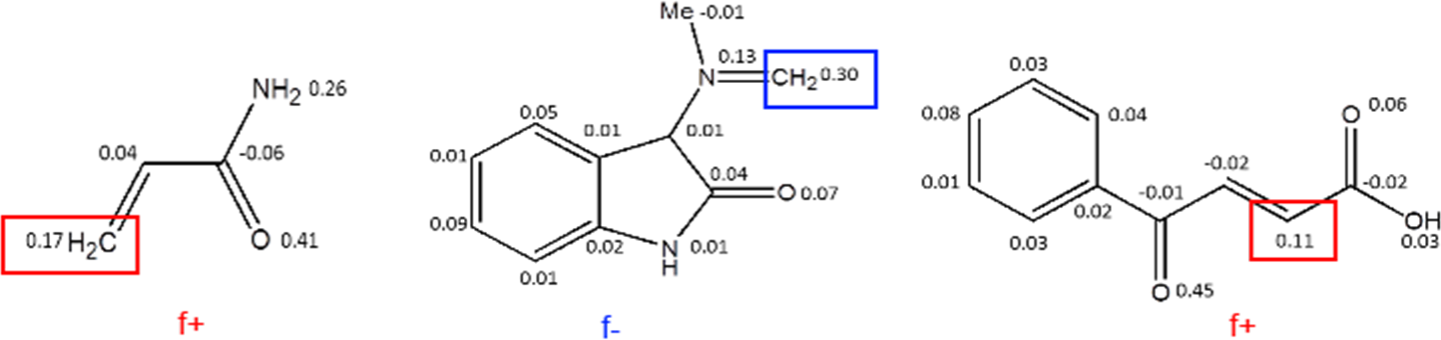
The Fukui function indices of acrylamide, azomethine ylide and benzoylacrylic acid.

The atom charges for the analysis of the Fukui function indices were calculated within the Natural Bonding Orbitals theory [40] with the NBO 5.0 program [41], that revealed the most reactive sites of the reagents. The reaction proceeds regioselectively with the addition of the most nucleophilic methylene group carbon of the azomethine ylide to the most electrophilic sites of the acrylamide and benzoylacrylic acid, which affords only one stereoisomer of cycloadducts **4** and **6** stereoselectively despite the presense of several stereocenters in the molecules (Figure 6).

**Scheme 2:**
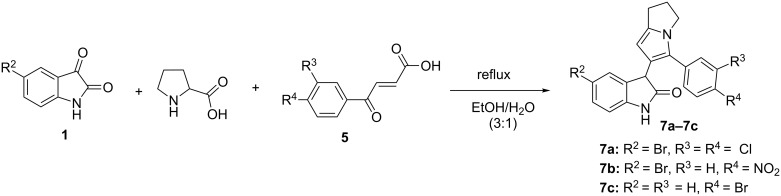
The synthesis of compounds **7a–7c**.

For assigning structures of byproducts we carried out the reaction of isatins **1**, aroylacrylic acids **5** and proline in a boiling mixture of EtOH and water, which resulted in the formation and isolation of compounds **7a–7c** (Scheme 2). The unexpected structure of rearranged product **7a** was confirmed by ^1^H, ^13^C and 2D NMR spectroscopy (Table 3).

**Table 3:** ^13^C and ^1^H spectral data for compound **7a**.

entry	functional group	^13^C	^1^H		
		δ, ppm	δ, ppm	multiplicity	*J*, Hz

1	1-NH	–	10.59	s	–
2	2-CO	178.09	–	–	–
3	3-CH	45.33	4.56	s	–
4	3a-C (oxindole)	134.06	–	–	–
5	4-CH (oxindole)	127.55	7.05	s	–
6	5-C (oxindole)	113.56	–	–	–
7	6-CH (oxindole)	130.77	7.33	dd	8.1; 2.2
8	7-CH (oxindole)	111.57	6.80	d	8.1
9	7a-C (oxindole)	142.21	–	–	–
10	5-C	124.67	–	–	–
11	6-C	120.38	–	–	–
12	7-CH	99.69	5.30	s	–
13	7a-C	138.34	–	–	–
14	1-CH_2_	24.43	2.85–2.63	m	–
15	2-CH_2_	27.31	2.43–2.27	m	–
16	3-CH_2_	46.26	4.20–4.02, 3.90–3.70	m	–
17	1-C Ar	129.50	–	–	–
18	2-CH Ar	129.87	7.82	d	1.8
19	3-C Ar	133.12	–	–	–
20	4-C Ar	131.63	–	–	–
21	5-CH Ar	130.95	7.65	d	8.1
22	6-CH Ar	128.44	7.55	dd	8.2; 1.8

**Figure 7:**
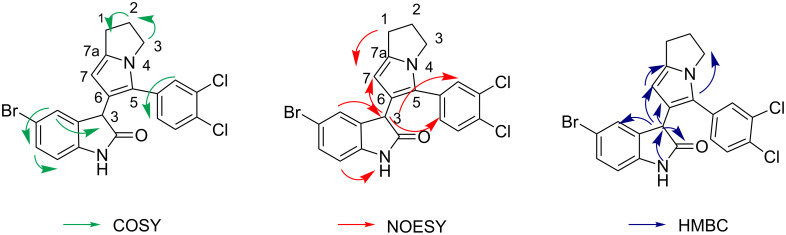
The selected COSY, NOESY and HMBC correlations of the signals in the ^1^H and ^13^C NMR spectra of compound **7a**.

The main feature of the ^13^C spectra of compounds **7a–7c** is the absence of the signal of the 3C-spiro nucleus. The ^1^H NMR spectrum of compound **7a** displays a singlet at 5.30 ppm for the 7-CH of the dihydropyrrolizinyl moiety, which shows a H,H-NOESY correlation with a singlet at 4.56 ppm (3-CH of the oxindole ring) and HMBCs with 7a-C at 138.34 ppm. The singlet at 4.56 ppm of 3-CH of the oxindole ring shows H,H-COSY and H,H-NOESY correlations with a singlet at 7.05 ppm of 4-CH (oxindole ring) and HMBCs with 2-CO at 178.09 ppm, 4-C at 127.55 ppm and 6-C at 120.38 ppm (Figure 7). The NH proton of the oxindole ring gives a singlet at 10.59 ppm.

**Scheme 3:**
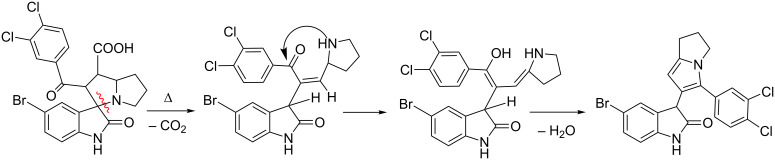
Tentative reaction mechanism for the decarboxylative cyclative rearrangement of the initial three-component product.

The tentative mechanism for the formation of **7a** is outlined in Scheme 3. First, the initially formed spiropyrrolizidine undergoes decarboxylation via ring opening of the spiro cycle. The subsequent enolization of the intermediate leads to the formation of the dihydropyrrolizinyl oxindole system.

## Conclusion

The 1,3-dipolar cycloaddition of azomethine ylides generated in situ from isatins and sarcosine or cyclic amino acids to acrylamides or aroylacrylic acids afforded regio- and stereoselectively the spirooxindoles **4** and **6** in moderate to good yields. The selectivity of the three-component condensation of isatins and α-amino acids with aroylacrylic acids can be controlled by the reaction temperature and the reaction medium. While spiro cycloadducts can be obtained in methanol dihydropyrrolizinyl oxindoles are formed in aqueous ethanol media at higher temperatures. Therefore, reactions involving aroylacrylic acids as substrates can afford the product in a regiocontrolled manner.

## Experimental

**Reagents and analytics:** The ^1^H NMR spectra were recorded on Varian Mercury VX-200 (200 MHz) and Bruker Avance DRX-500 (500 MHz) instruments in DMSO-*d*_6_ with TMS as an internal standard. The ^13^C NMR spectra were recorded on a Bruker Avance DRX-500 (125 MHz) and Bruker AM-300 (75 MHz) instruments in DMSO-*d*_6_ with TMS as an internal standard. The COSY, NOESY, HSQC, and HMBC spectra were recorded using the standard procedure with gradient separation of the signal. The mass spectra were recorded on a Varian 1200L GC–MS instrument, ionization by EI at 70 eV. Elemental analysis was carried out on an EA 3000 Eurovector elemental analyzer. Melting points were determined on a Kofler hot bench. The progress of reactions and also the purity of the obtained compounds were monitored by TLC on Silufol UV-254 plates with acetone/heptane (4:1) as an eluent. Commercially available reagents and solvents were used without further purification. The aroylacrylic acids **5** were prepared according to the previously reported procedure [42].

**General procedure for the synthesis of spirooxindoles 4a–4g from the three-component reaction of isatins, sarcosine or cyclic α-amino acids and acrylamides:** A mixture of isatin (1.0 mmol), α-amino acid (1.0 mmol) and acrylamide (1.0 mmol) in 4.0 mL aqueous methanol (1:3) was heated in an oil bath to reflux temperature for 40 min to 7 hours. The resulting precipitates were collected by filtration and washed with cold methanol to give the analytically pure products **4**. **4a:** colorless solid, 31%, mp 260–262 °C; ^1^H NMR (200 MHz, DMSO-*d_6_*) δ 10.42 (s,1H, 1-NH), 7.32 (dd, *J* = 8.2, 1.8 Hz, 1H, 6-CH), 7.21 (d, *J* = 1.8 Hz, 1Н, 4-СН), 7.11 (s, 1Н, NH-amide), 6.74 (s, 1Н, NH-amide), 6.70 (d, *J* = 8.1 Hz, 1Н, 7-СН), 3.11–2.99 (m, 2Н, 4’-СН_2_), 2.99–2.89 (m, 1Н, 3’-СН), 2.38–2.26 (m, 1Н, 5’-СН_2_), 2.13–1.98 (m, 1Н, 5’-СН_2_), 1.91 (s, 3Н, 1’-NСН_3_); ^13^C NMR (75 MHz, DMSO-*d*_6_) δ 178.03 (2-CO), 170.35 (CONH_2_), 142.38, 131.75, 129.77, 127.23, 112.27, 111.06, 72.26 (C-spiro), 56.51, 48.56, 34.38, 25.44; MS (*m*/*z*) (%): 325/323 (M^+^, 19/20), 295/292 (59/78), 280/278 (97/100), 252/250 (54/38), 131/129 (15/43), 57 (78); anal. calcd for C_13_H_14_BrN_3_O_2_ (324.17): C 48.17, H 4.35, N 12.96; found: C 48.19, H 4.40, N 12.99.

**General procedure for the synthesis of spirooxindoles 6a–6h from the three-component reaction of isatins, sarcosine or proline and aroylacrylic acids:** A mixture of isatin (1.0 mmol), α-amino acid (1.0 mmol) and aroylacrylic acid (1.0 mmol) in 4.0 mL aqueous methanol (1:3) was heated in an oil bath to reflux temperature for about 20 min or stirred at room temperature for 25 min to 12 hours. The resulting precipitates were collected by filtration and washed with cold methanol to give the analytically pure products **6**. **6a:** colorless solid, 50%, mp 240–242 °C; ^1^H NMR (200 MHz, DMSO-*d*_6_) δ 12.81 (s, 1H, 4’-COOH), 10.65 (s, 1H, 1-NH), 7.63 (d, *J* = 8.4 Hz, 1H, 5-CH (dichlorobenzoyl)), 7.44 (s, 1H, 2-CH (dichlorobenzoyl)), 7.36 (d, *J* = 8.4 Hz, 1H, 6-CH (dichlorobenzoyl)), 7.21 (d, *J* = 8.1 Hz, 1H, 6-CH), 6.96 (s, 1H, 4-CH), 6.44 (d, *J* = 8.4 Hz, 1H, 7-CH), 4.51 (d, *J* = 9.2 Hz, 1H, 3’-CH), 3.99 (q, *J* = 8.4 Hz, 1H, 4’-CH), 3.32–3.12 (m, 2H, 5’-CH_2_), 1.96 (s, 3H, 1’-NCH_3_); ^13^C NMR (125 MHz, DMSO-*d*_6_) δ 195.09 (CO-benzoyl), 177.42 (2-CO), 173.16 (4’-COOH), 141.26, 136.44, 132.08, 131.70, 130.95, 130.32, 129.02, 128.55, 128.20, 127.13, 113.55, 111.33, 72.27 (C-spiro), 56.54, 54.66, 42.96, 34.40; anal. calcd for C_20_H_15_BrСl_2_N_2_O_4_ (498.15): C 48.22, H 3.04, N 5.62; found: C 48.17, H 3.10, N 5.67.

**General procedure for synthesis of compounds 7a–7c from the three-component reaction of isatins, proline and aroylacrylic acids:** A mixture of isatin (1.0 mmol), proline (1.0 mmol) and aroylacrylic acid (1.0 mmol) in 4.0 mL aqueous ethanol (1:3) was heated in an oil bath to reflux temperature for 15 min. The resulting precipitates were collected by filtration and washed with cold ethanol to give analytically pure products **7**. **7a**: orange powder, 22%, mp 215–216 °C. ^1^H (500 MHz, DMSO-*d*_6_) and ^13^C NMR (125 MHz, DMSO-*d*_6_) data are given in Table 3. MS (*m*/*z*) (%): 462 (M^+^, 52), 435 (45), 405 (8), 353 (12), 317 (18), 289 (52), 208 (30), 173 (33), 127 (46), 75 (30), 41 (100); anal. calcd for C_21_H_15_BrСl_2_N_2_O (462.17): C 54.57; H 3.27; N 6.06; found: C 54.48; H 3.19; N 6.10.

### Experimental part of X-ray diffraction study

The colourless crystals of **4a** (C_13_H_14_N_3_O_2_Br) are triclinic. At 293 K, *a* = 6.9659(3), *b* = 7.5441(4), *c* = 13.4092(5) Å, α = 90.994(4)°, β = 90.947(4)°, γ = 106.218(5)°, *V* = 676.36(5) Å^3^, *M*_r_ = 324.18, *Z* = 2, space group P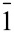, *d*_calc_ = 1.592 g/сm^3^, μ(Mo K_α_) = 3.040 mm^−1^, F(000) = 328. Intensities of 6411 reflections (3942 independent, *R*_int_ = 0.018) were measured on an «Xcalibur-3» diffractometer (graphite monochromated Mo K_α_ radiation, CCD detector, ω-scaning, 2Θ_max_ = 60°).

The colourless crystals of **6a** (C_21_H_19_N_2_O_5_BrCl_2_) are triclinic. At 293 K, *a* = 8.8751(7), *b* = 10.5764(9), *c* = 12.352(1) Å, α = 75.858(5)°, β = 84.297(5)°, γ = 76.237(5)°, *V* = 1091.0(2) Å^3^, *M*_r_ = 530.19, *Z* = 2, space group P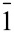, *d*_calc_= 1.614 g/сm^3^, μ(Mo K_α_) = 2.165 mm^−1^, F(000) = 536. Intensities of 15460 reflections (3842 independent, *R*_int_ = 0.043) were measured on an «Xcalibur-3» diffractometer (graphite monochromated Mo K_α_ radiation, CCD detector, ω-scaning, 2Θ_max_ = 50°).

The structures were solved by direct methods using the SHELXTL package [43]. The absorption correction was performed using the multi-scan method (*T*_min_ = 0.582, *T*_max_ = 0.751 for **4a** and *T*_min_ = 0.563 *T*_max_ = 0.671 for **6a**). Position of the hydrogen atoms were located from electron density difference maps and refined by “riding” model with U_iso_ = nU_eq_ of the carrier atom (*n* = 1.5 for methyl and hydroxy groups and *n* = 1.2 for other hydrogen atoms). Full-matrix least-squares refinement of the structures against F^2^ in anisotropic approximation for non-hydrogen atoms using 3908 (**4a**), 3801 (**6a**) reflections was converged to: wR_2_ = 0.111 (R_1_ = 0.046 for 2795 reflections with F>4σ(F), S = 1.049) for structure **4a** and wR_2_ = 0.110 (R_1_ = 0.043 for 2435 reflections with F>4σ(F), S = 1.055) for structure **6a**. The final atomic coordinates, and crystallographic data for molecules **4a** and **6a** have been deposited at the Cambridge Crystallographic Data Centre, 12 Union Road, CB2 1EZ, UK (fax: +44-1223-336033; e-mail: deposit@ccdc.cam.ac.uk) and are available on request quoting the deposition numbers CCDC 964021 for **4a** and CCDC 972494 for **6a**).

## Supporting Information

File 1 Spectroscopic and analytical data.

File 2 X-ray diffraction data description for compounds **4a** and **6a**.

File 3 Crystallographic information file for compound **4a**.

File 4 Crystallographic information file for compound **6a**.
