# Supplementary material for: The regioselective synthesis of spirooxindolo pyrrolidines and pyrrolizidines via three-component reactions of acrylamides and aroylacrylic acids with isatins and α-amino acids
Source: Beilstein J Org Chem. 2014 Jan 9;10:117–26. doi: 10.3762/bjoc.10.8 (PMC3896290; doi:10.3762/bjoc.10.8)
Supplement: File 1 — Spectroscopic and analytical data. [file Beilstein_J_Org_Chem-10-117-s001.pdf]

Supporting Information

for

The regioselective synthesis of spirooxindolo pyrrolidines and pyrrolizidines via three-component reactions of acrylamides and aroylacrylic acids with isatins and α-amino acids

Tatyana L. Pavlovskaya[1], Fedor G. Yaremenko[1,2], Victoria V. Lipson*[1,2,3], Svetlana V. Shishkina[1], Oleg V. Shishkin[1,3], Vladimir I. Musatov[1], Alexander S. Karpenko[4]

Address:[1]State Scientific Institution "Institute for Single Crystals" of National Academy of Sciences of Ukraine, 60, Lenin ave., Kharkov, 61178, Ukraine, [2]Antidiabetic Drug Laboratory, State Institution "V.J. Danilevsky Institute of Problems of Endocrine Pathology at the Academy of Medical Sciences of Ukraine", 10, Artem St., Kharkov, 61002, Ukraine, [3]Organic Chemistry Department, V.N. Karazin Kharkov National University, 4, Svobody Sq., 61077, Kharkov, Ukraine, and [4]A.V. Bogatsky physico-chemical institute of the National Academy of Sciences of Ukraine, 86, Lustdorfskaya doroga, 65080, Odessa, Ukraine

Email: Victoria V. Lipson* - lipson@ukr.net

*Corresponding author

Spectroscopic and analytical data

Table of contents

Reagents and analytics ……………………………………………………………………..S2

Synthetic procedures and characterization data for compounds 4a–4g…………………S2

Synthetic procedures and characterization data for compounds 6a–6h ……………….. S11

Synthetic procedure, characterization data for compounds 7a–7c ……………...………S20

Reagents and analytics: The ^{1}H NMR spectra were recorded on Varian Mercury VX-200 (200 MHz) and Bruker Avance DRX-500 (500 MHz) instruments in DMSO-d_6 with TMS as an internal standard. The ^{13}C NMR spectra were recorded on a Bruker Avance DRX-500 (125 MHz) and Bruker AM-300 (75 MHz) instruments in DMSO-d_6 with TMS as an internal standard. The COSY, NOESY, HSQC, and HMBC spectra were recorded using the standard procedure with gradient separation of the signal. The mass spectra were recorded on a Varian 1200L GC–MS instrument, ionization by EI at 70 eV. Elemental analysis was carried out on an EA 3000 Eurovector elemental analyzer. Melting points were determined on a Kofler hot bench. The progress of reactions and also the purity of the obtained compounds were monitored by TLC on Silufol UV-254 plates with acetone–heptane (4:1) as an eluent. Commercially available reagents and solvents were used without further purification.

General procedure for the synthesis of spirooxindoles 4a–4g from the three-component reaction of isatins, sarcosine or cyclic α-amino acids and acrylamides: A mixture of isatin (1.0 mmol), α-amino acid (1.0 mmol) and acrylamide (1.0 mmol) in 4.0 mL aqueous methanol (1:3) was heated in an oil bath to reflux temperature for 40 min to 7 hours. The resulting precipitates were collected by filtration and washed with cold methanol to give the analytically pure products **4**.

5-Bromo-1'-methyl-2-oxo-1,2-dihydrospiro[indole-3,2'-pyrrolidine]-3'-carboxamide (4a): colorless solid, 31%, mp 260-262 °C; ^{1}H NMR (200 MHz, DMSO-d_6) δ: 10.42 (s,1H, 1-NH), 7.32 (dd, *J*=8.2, 1.8 Hz, 1H, 6-CH), 7.21 (d, *J*=1.8 Hz, 1H, 4-CH), 7.11 (s, 1H, NH-amide), 6.74 (s, 1H, NH-amide), 6.70 (d, *J*=8.1 Hz, 1H, 7-CH), 2.99-3.11 (m, 2H, 4'-CH_2), 2.89-2.99 (m, 1H, 3'-CH), 2.26-2.38 (m, 1H, 5'-CH_2), 1.98-2.13 (m, 1H, 5'-CH_2), 1.91 (s, 3H, 1'-NCH_3); ^{13}C NMR (75 MHz, DMSO-d_6) δ: 178.03 (2-CO),170.35 ($CONH_2$), 142.38, 131.75, 129.77, 127.23, 112.27, 111.06, 72.26 (C-spiro), 56.51, 48.56, 34.38, 25.44; MS (m/z) (%):325/323 (M^+, 19/20),

295/292 (59/78), 280/278 (97/100), 252/250 (54/38), 131/129 (15/43), 57 (78). Anal. calcd. for $C_{13}H_{14}BrN_3O_2$ (324.17): C 48.17, H 4.35, N 12.96; Found: C 48.19, H 4.40, N 12.99.

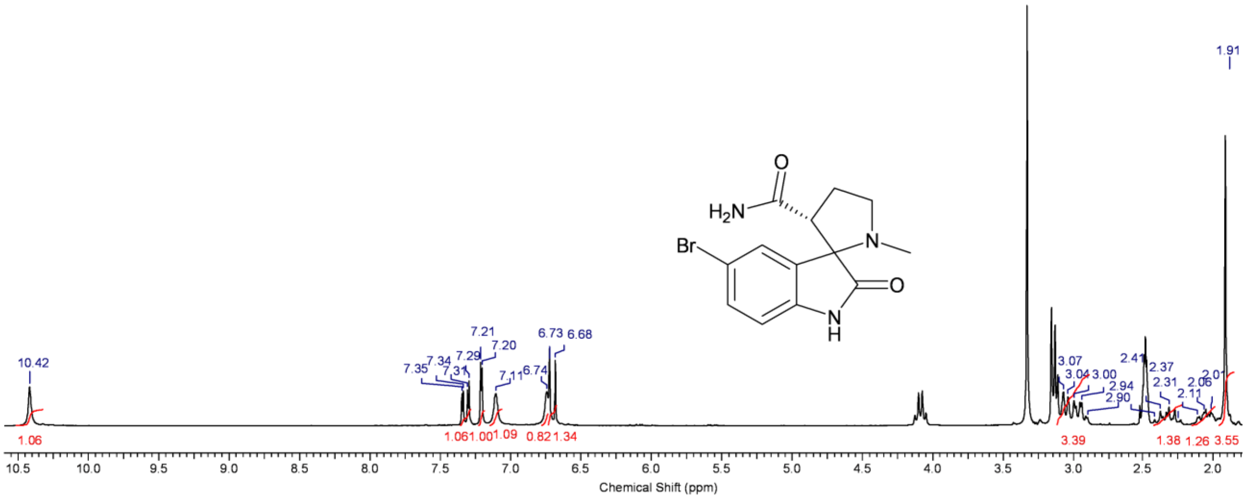

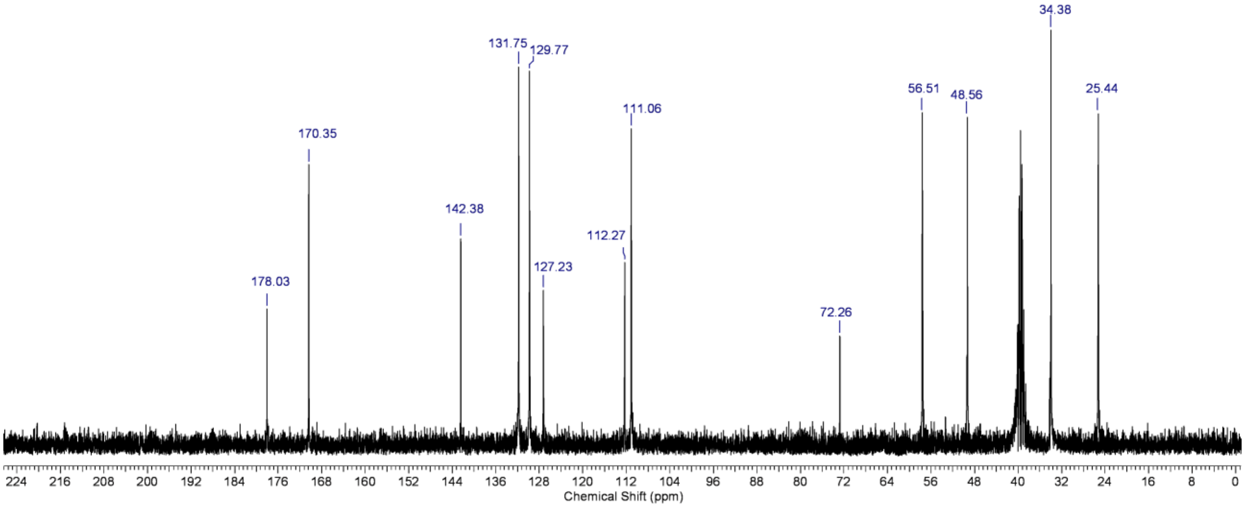

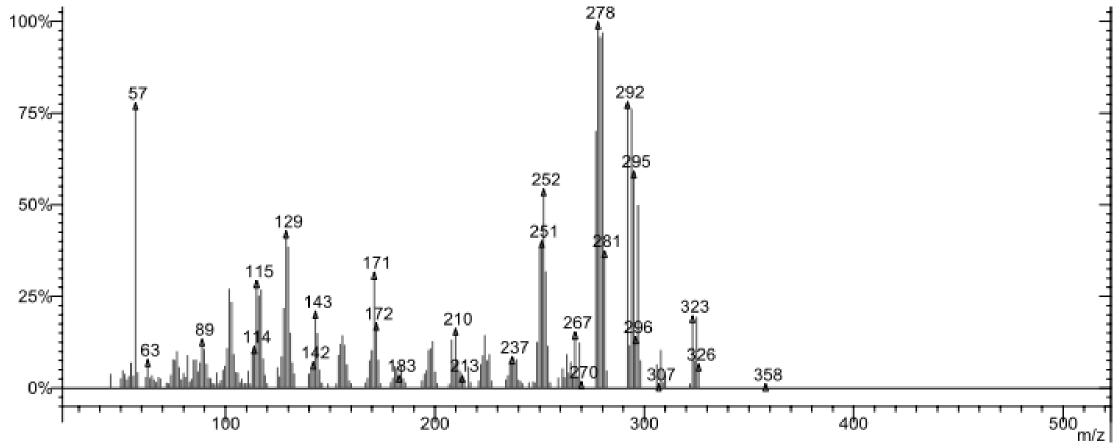

5-Nitro-2-oxo-1,1',2,2',5',6',7',7a'-octahydrospiro[indole-3,3'-pyrrolizine]-2'-carboxamide (4b): yellow powder, 85%, mp 220-222 °C; ^{1}H NMR (200 MHz, DMSO-d_6) δ: 10.96 (s,1H, 1-NH), 8.16 (dd, *J*=8.6, 2.1 Hz, 1H, 6-CH), 7.91 (d, *J*=2.1 Hz, 1H, 4-CH), 7.13 (s, 1H, NH-amide), 6.95 (d, *J*=8.6 Hz, 1H, 7-CH), 6.82 (s, 1H, NH-amide), 3.92-3.72 (m, 1H, 7a'-

CH), 3.49-3.35 (m, 1H, 2’-CH), 2.41-2.23 (m, 4H, 7’-CH_2, 1’-CH_2), 2.08-1.85 (m, 2H, 6’-CH_2), 1.84-1.36 (m, 2H, 5’-CH_2); ^{13}C NMR (75 MHz, DMSO-d_6) δ: 179.08 (2-CO), 170.36 ($CONH_2$), 149.85, 141.01, 126.52, 125.67, 122.66, 109.57, 73.06 (C-spiro), 66.98, 56.41, 47.10, 37.35, 30.75, 26.83. Anal. calcd. for $C_{15}H_{16}N_4O_4$ (316.31):C 56.96; H 5.10; N 17.71; Found: C 54.96; H 6.40; N 16.99.

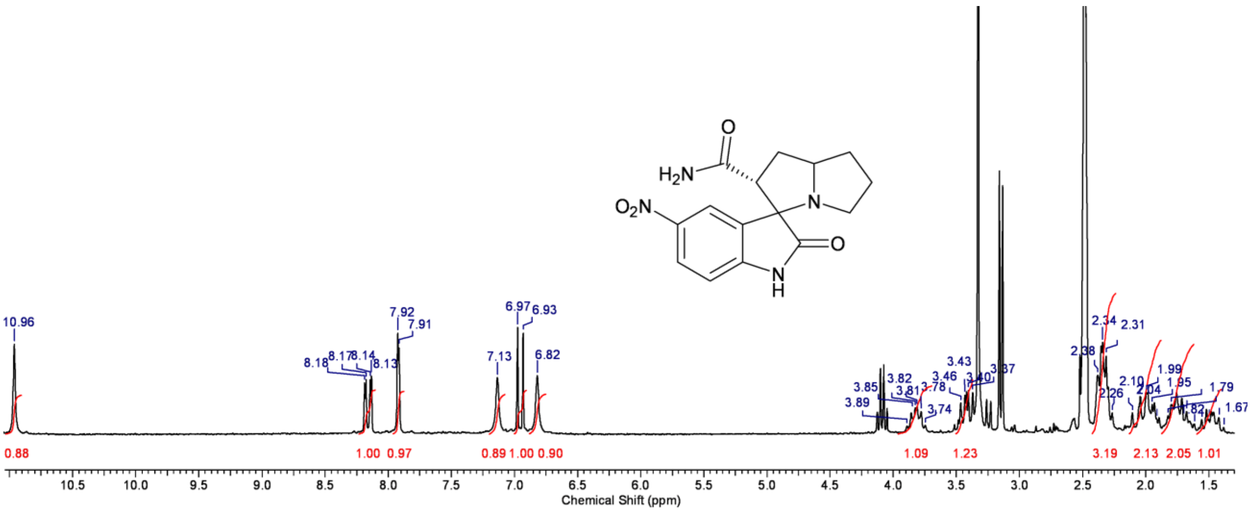

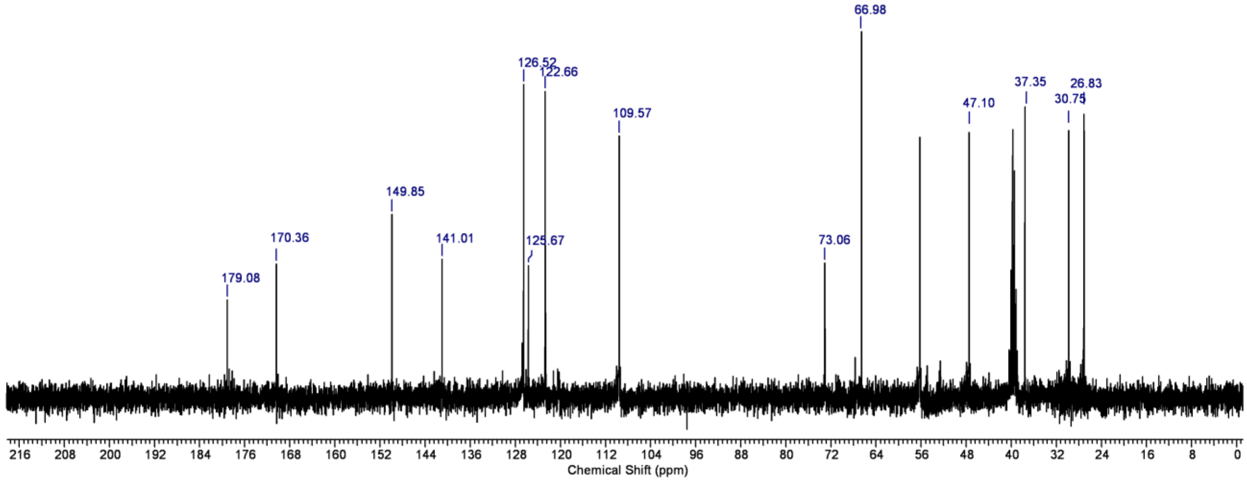

NOE

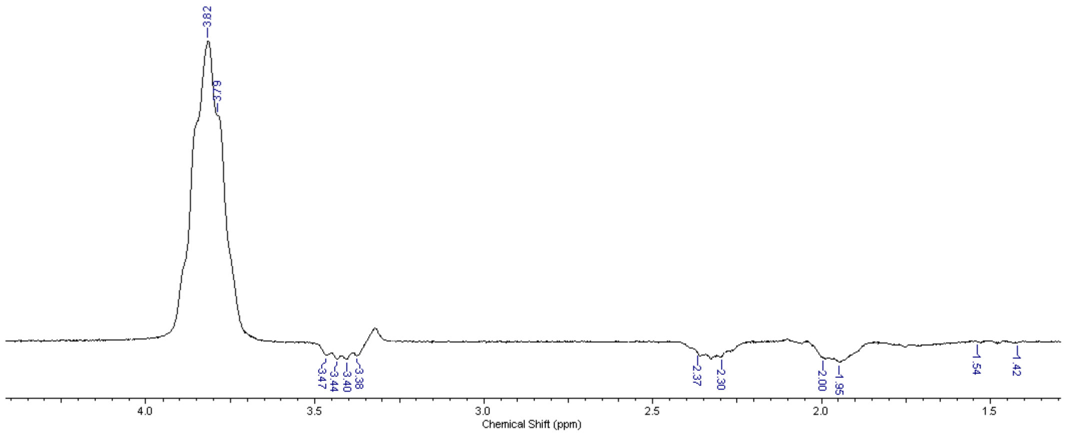

2'-Methyl-5-nitro-2-oxo-1,1',2,2',5',6',7',7a'-octahydrospiro[indole-3,3'-pyrrolizine]-2'-carboxamide (4c): colorless solid, 37%, mp 260-262 °C; ^{1}H NMR (200 MHz, DMSO-d_6) δ: 10.86 (s,1H, 1-NH), 8.12 (d, J=8.5 Hz, 1H, 6-CH), 7.89 (s, 1H, 4-CH), 7.04 (s, 1H, NH-amide), 6.88 (d, J=8.5 Hz, 1H, 7-CH), 6.74 (s, 1H, NH-amide), 3.93-3.78 (m, 1H, 7a'-CH), 2.41-2.31 (m, 2H, 5'-CH_2), 2.12-2.02 (m, 3H, 6'-CH_2, 1'-CH_2), 1.91-1.64 (m, 2H, 7'-CH_2), 1.51-1.25 (m, 4H, 2'-CH_3, 6'-CH_2); ^{13}C NMR (75 MHz, DMSO-d_6) δ: 179.08 (2-CO), 170.36 ($CONH_2$), 149.85, 141.01, 126.52, 125.67, 122.66, 109.57, 73.86 (C-spiro), 64.98, 58.44, 48.83, 37.35, 30.75, 26.83, 22.34. Anal. calcd. for $C_{16}H_{18}N_4O_4$ (330.34): C 58.17; H 5.49; N 16.96; Found: C 57.90; H 5.80; N 16.20.

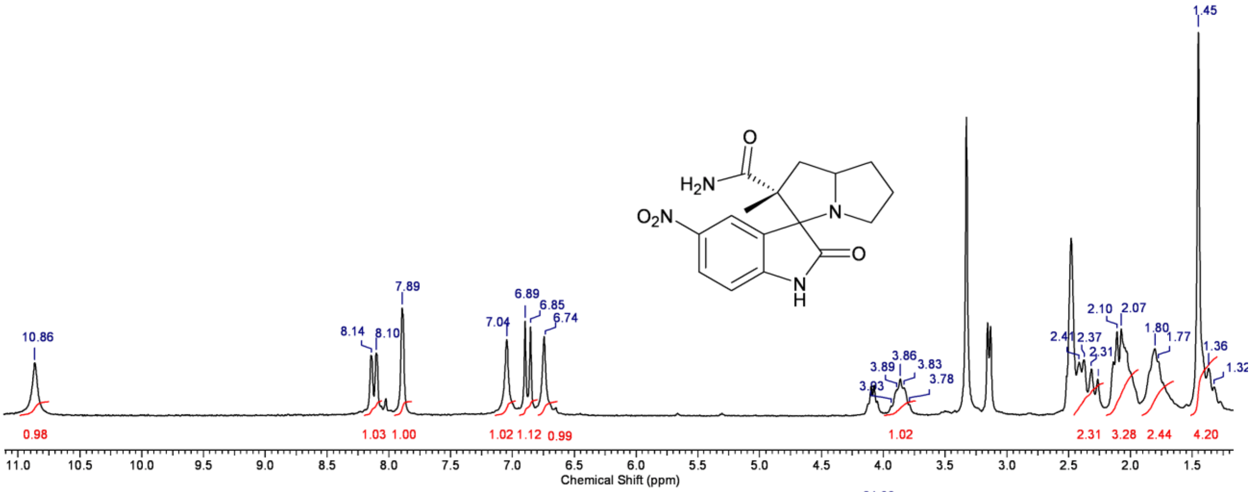

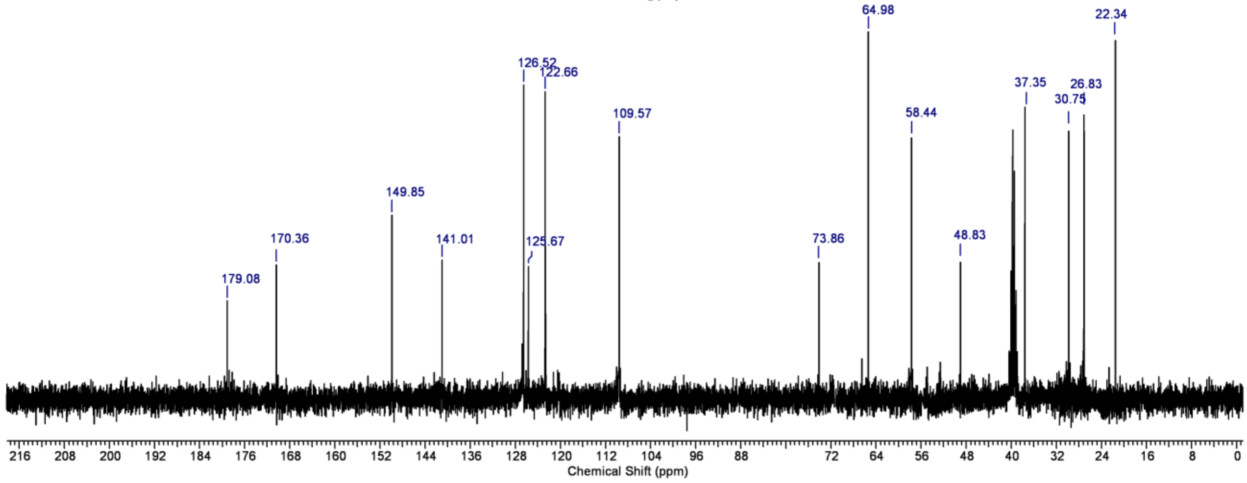

NOE

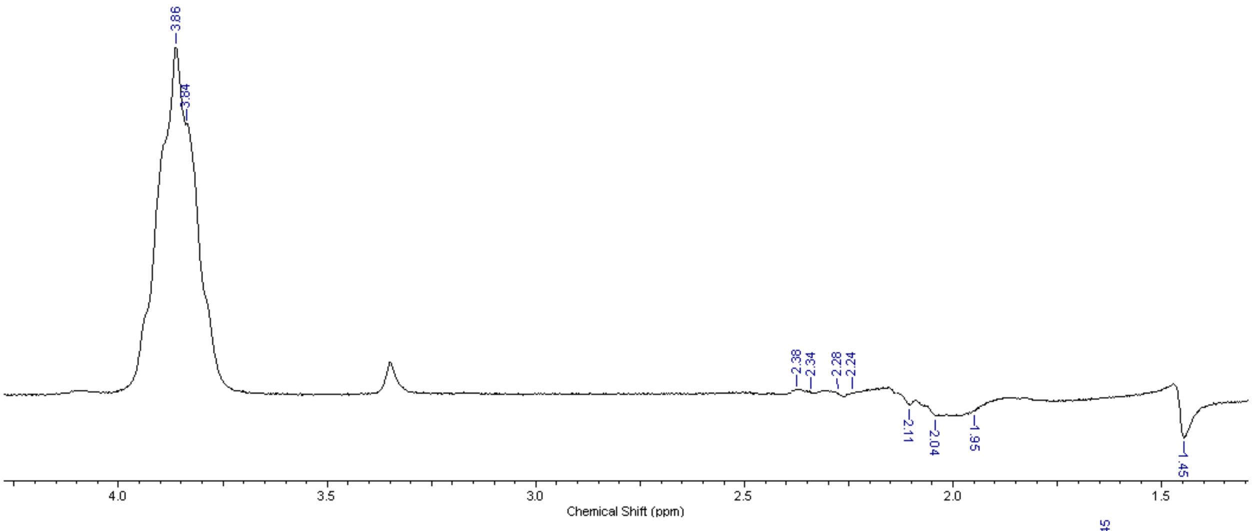

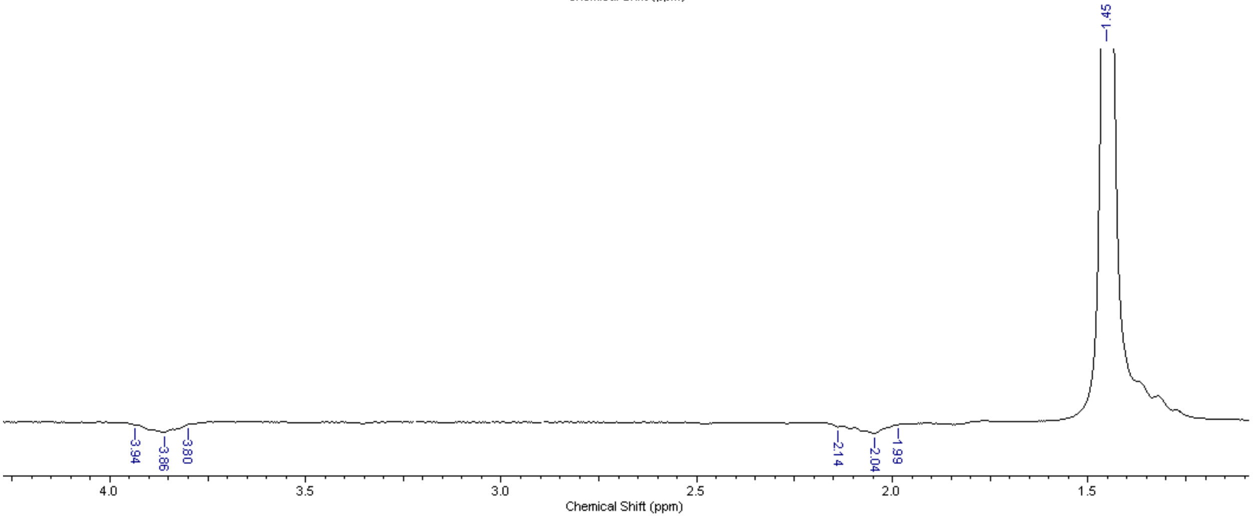

5-Bromo-2-oxo-1,1',2,6',7',7a'-hexahydrospiro[indole-3,5'-pyrrolo[1,2-*c*][1,3]thiazole]-6'-carboxamide (4d): white powder, 42%, mp 340-342 °C; ^{1}H NMR (200 MHz, DMSO-d_6) δ: 10.44 (s, 1H, 1-NH), 7.40 (s, 1H, 4-CH), 7.33 (d, *J*=8.2 Hz, 1H, 6-CH), 7.03 (s, 1H, NH-amide), 6.79 (s, 1H, NH-amide), 6.71 (d, *J*=8.2 Hz, 1H, 7-CH), 4.07-3.90 (m, 1H, 7a'-CH), 3.63 (d, 1H, 3'-CH_2, ^{2}J=10.1 Hz), 3.25-2.95 (m, 2H, 3'-CH_2, 6'-CH), 2.94-2.69 (m, 2H, 1'-CH_2), 2.40-1.90 (m, 2H, 7'-CH_2);^{13}C NMR (75 MHz, DMSO-d_6) δ: 178.06 (2-CO), 170.37 ($CONH_2$), 142.40, 131.78, 129.80, 127.25, 112.29, 111.09, 73.13 (C-spiro), 67.95, 54.59, 53.35, 37.14, 33.02. Anal. calcd. for $C_{14}H_{14}BrN_3O_2S$ (368.25): C 45.66; H 3.83; N 11.41; S 8.71; Found: C 45.50; H 4.30; N 11.53; S 8.65.

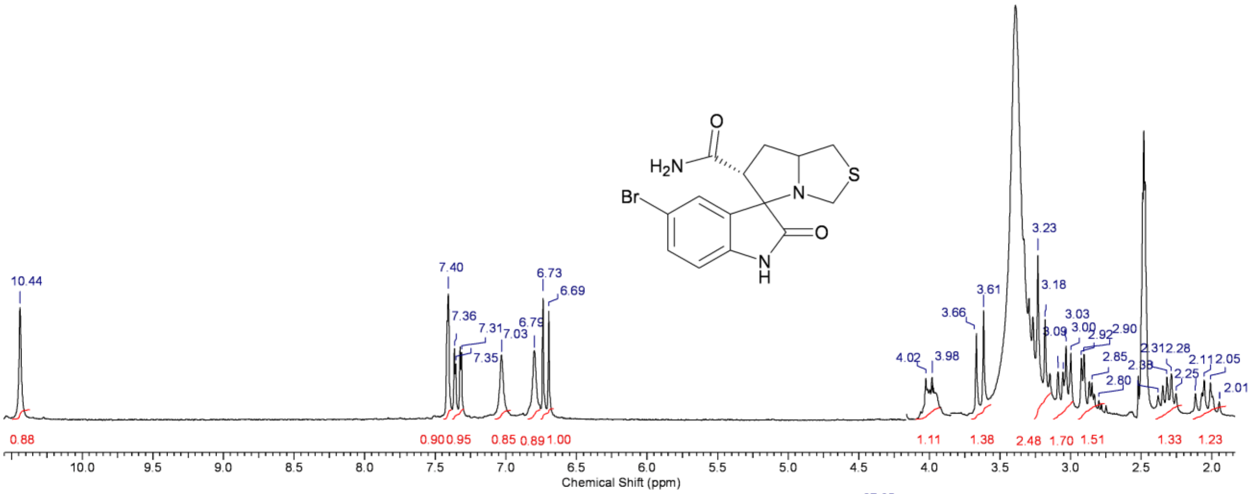

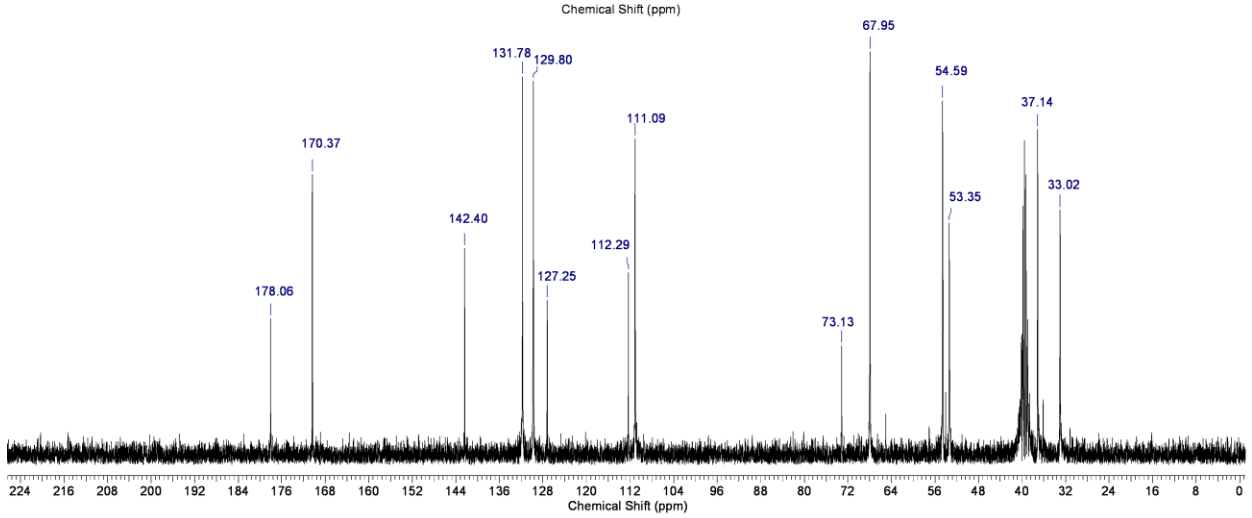

NOE

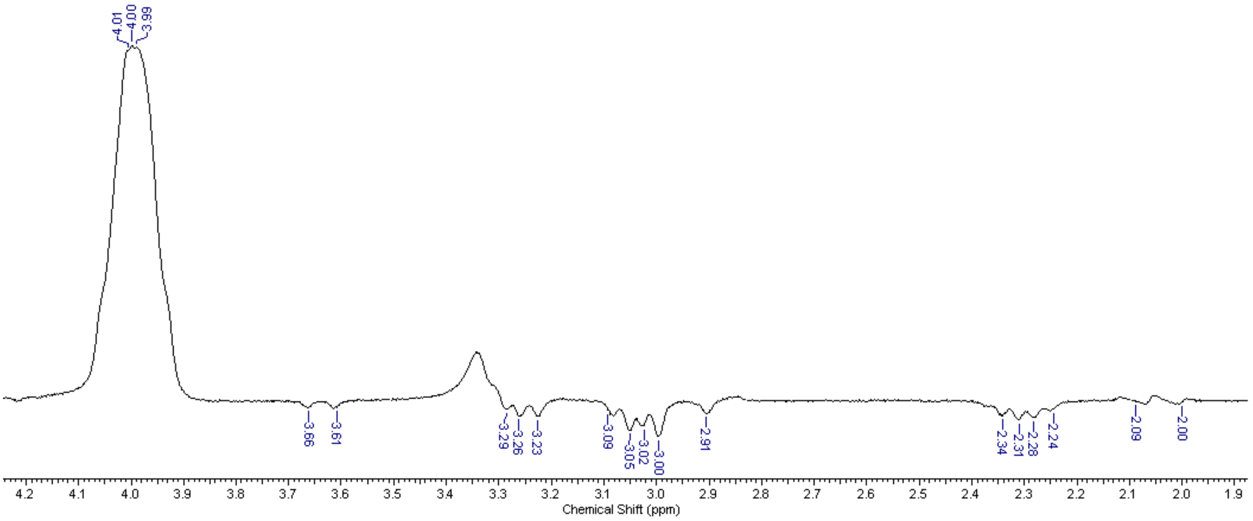

5-Nitro-2-oxo-1,1',2,6',7',7a'-hexahydrospiro[indole-3,5'-pyrrolo[1,2-*c*][1,3]thiazole]-6'-carboxamide (4e): brown powder, 60%, mp 255-257 °C; ^{1}H NMR (200 MHz, DMSO-d_6) δ: 11.06 (s, 1H, 1-NH), 8.41-7.96 (m, 2H, 4-CH, 6-CH), 7.17 (s, 1H, NH-amide), 7.04-6.78 (m, 2H, 7-CH, NH-amide), 4.20-3.95 (m, 1H, 7a'-CH), 3.66 (d, 1H, 3'-CH_2, ^{2}J=10.7 Hz), 3.30-2.87 (m,

4H, 3’-CH_2, 6’-CH, 1’-CH_2), 2.43-1.90 (m, 2H, 7’-CH_2); ^{13}C NMR (75 MHz, DMSO-d_6) δ: 179.08 (2-CO), 170.34 ($CONH_2$), 149.85, 141.01, 126.52, 125.67, 122.66, 109.56, 73.06 (C-spiro), 68.08, 54.88, 53.86, 37.35, 33.27. Anal. calcd. for $C_{14}H_{14}N_4O_4S$ (334.35): C 50.29; H 4.22; N 16.76; S 9.59; Found: C 50.31; H 4.24; N 16.53; S 9.65.

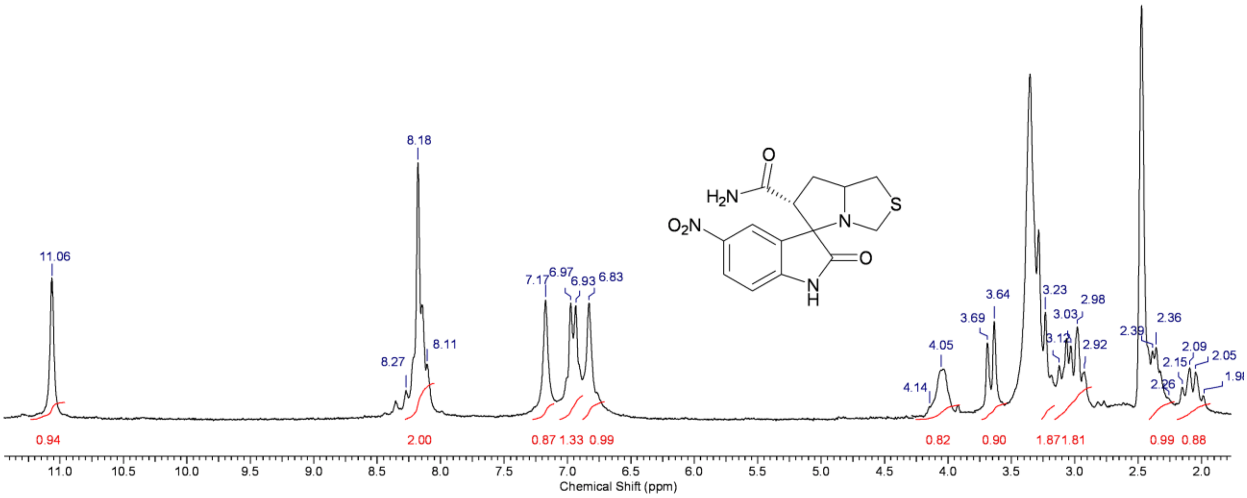

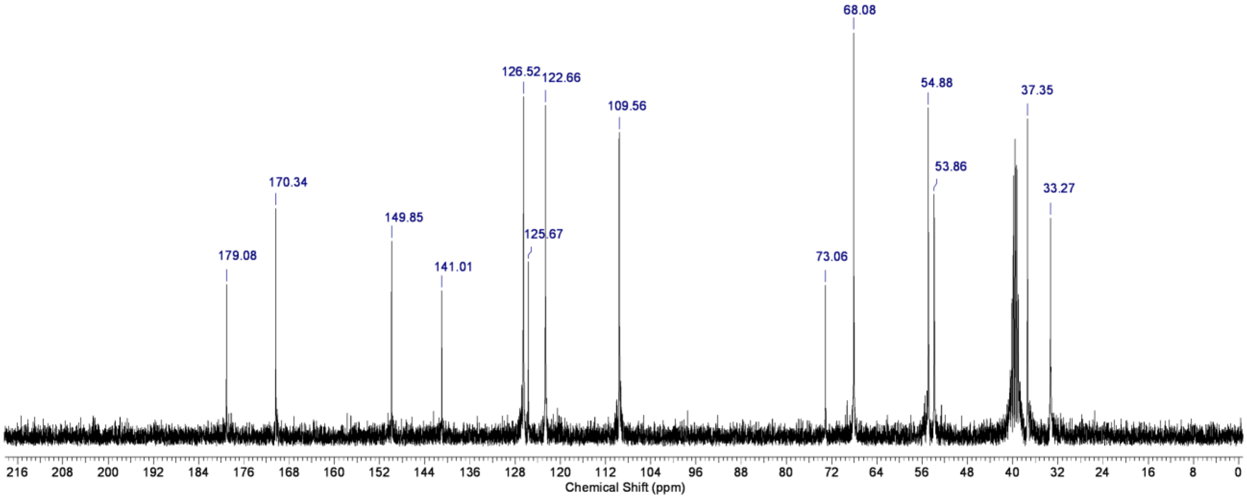

1-(4-Chlorobenzyl)-2-oxo-1,1',2,6',7',7a'-hexahydrospiro[indole-3,5'-pyrrolo[1,2-*c*][1,3]thiazole]-6'-carboxamide (4f): white solid, 38%, mp 242-244 °C; ^{1}H NMR (200 MHz, DMSO-d_6) δ: 7.41-7.25 (m, 5H, $CH_2\underline{C_6H_4}Cl$, 4-CH), 7.15 (t, *J*=7.2 Hz, 1H, 5-CH) 7.04 (s, 1H, NH-amide), 6.91 (t, *J*=6.9 Hz, 1H, 6-CH), 6.81 (s, 1H, NH-amide), 6.70 (d, *J*=6.9 Hz, 1H, 7-CH), 5.10-4.75 (m, 2H, $\underline{CH_2}C_6H_4Cl$), 4.15-4.00 (m, 1H, 7a’-CH), 3.66 (d, ^{2}J=10.7 Hz, 1H, 3’-CH_2), 3.50-3.38 (m, 1H, 6’-CH), 3.17 (d, ^{2}J=10.7 Hz, 1H, 3’-CH_2), 3.12-2.79 (m, 2H, 1’-CH_2), 2.43-2.10 (m, 2H, 7’-CH_2).^{13}C NMR (75 MHz, DMSO-d_6) δ: 178.84 (2-CO), 170.36 ($CONH_2$), 141.81, 135.49, 131.48, 129.40, 129.32, 127.22, 127.05, 124.30, 121.24, 109.69, 73.04 (C-spiro),

67.95, 56.01, 54.59, 53.35, 36.11, 32.99; MS (m/z) (%):413 (M^+, 5), 367 (5), 313 (6), 296 (10), 241 (4), 197 (10), 146 (8), 125 (100), 89 (13), 44 (7). Anal. calcd. for $C_{21}H_{20}ClN_3O_2S$ (413.92): C 60.94; H 4.87; N 10.15; S 7.75; Found: C 61.02; H 4.76; N 10.17; S 7.69.

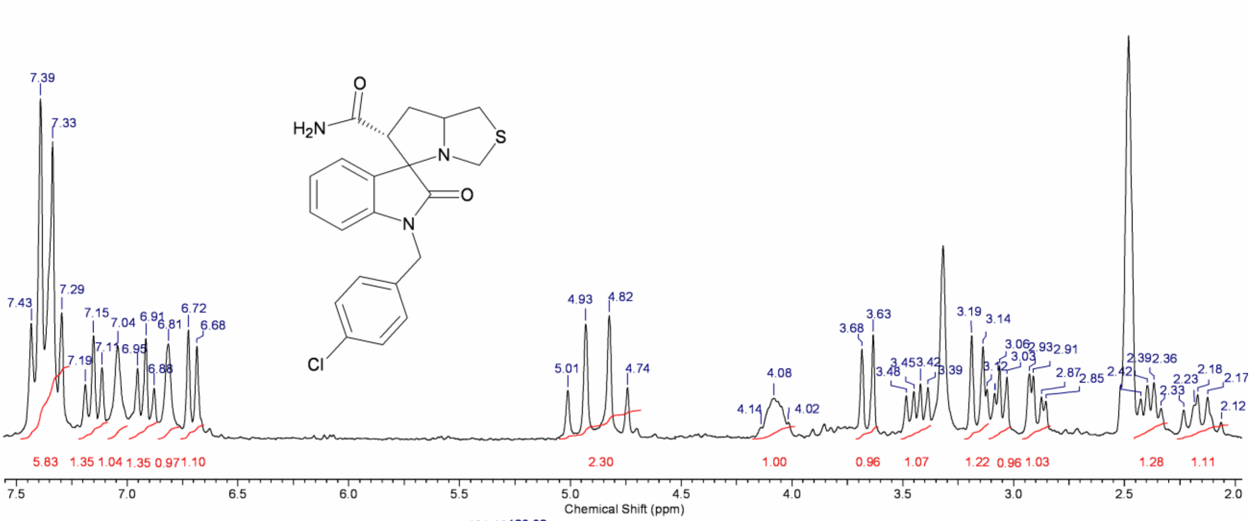

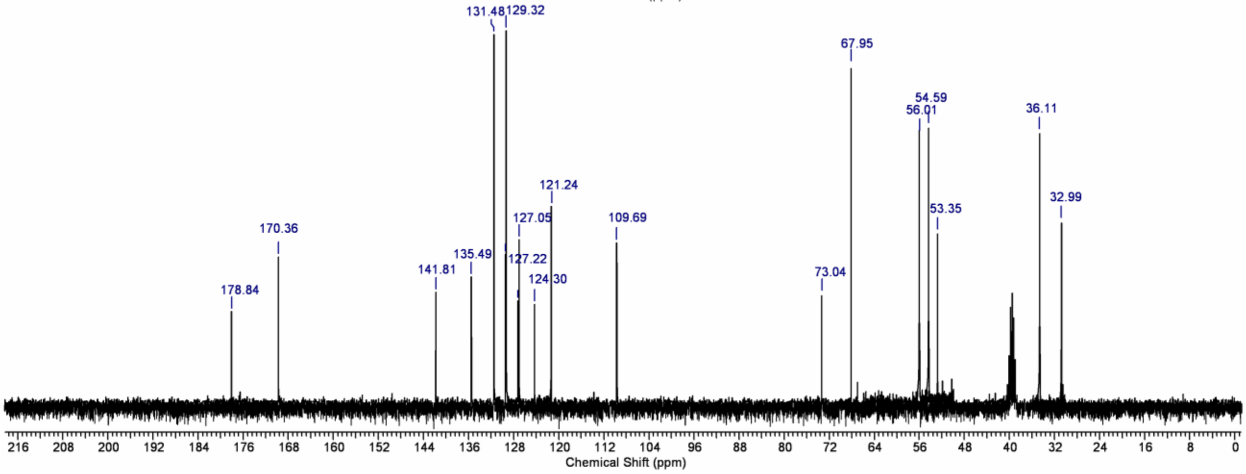

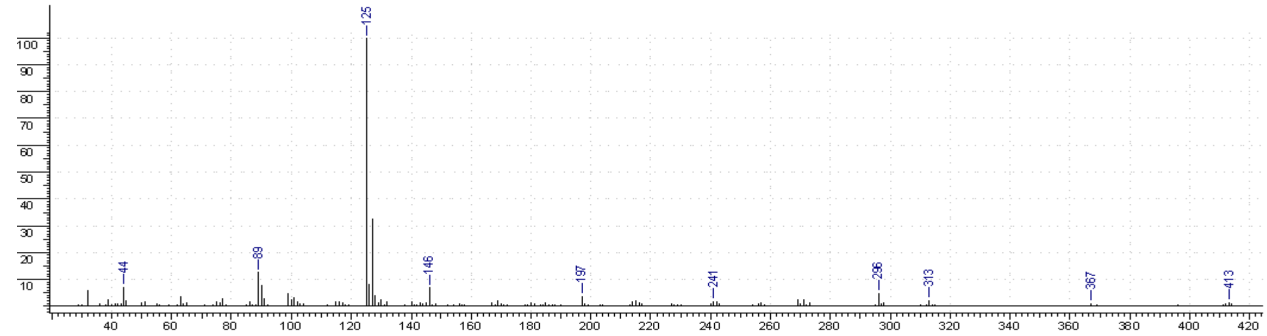

5-Bromo-1,2'-dimethyl-2-oxo-1,1',2,2',5',6',7',7a'-octahydrospiro[indole-3,3'-pyrrolizine]-2'-carboxamide (4g): colorless solid, 58%, mp 248-249 °C; ^{1}H NMR (200 MHz, DMSO-d_6) δ: 7.39 (dd, J=8.2, 1.8 Hz, 1H, 6-CH), 7.21 (d, J=1.8 Hz, 1H, 4-CH), 6.90-6.80 (m,

2H, NH-amide, 7-CH), 6.64 (s, 1H, NH-amide), 3.92-3.71 (m, 1H, 7a'-CH), 3.03 (s, 3H, 1-NCH_3), 2.38-2.23 (m, 2H, 5'-CH_2), 2.12-1.71 (m, 5H, 6'-CH_2, 1'-CH_2, 7'-CH_2), 1.51-1.29 (m, 4H, 2'-CH_3, 6'-CH_2); ^{13}C NMR (75 MHz, DMSO-d_6) δ: 178.06 (2-CO), 170.38 ($CONH_2$), 142.40, 131.78, 129.79, 127.25, 112.29, 111.08, 73.15 (C-spiro), 64.98, 58.46, 48.82, 37.14, 31.22, 30.75, 26.83, 22.36; MS (m/z) (%):378 (M^+, 5), 333 (4), 292 (90), 264 (10), 213 (14), 184 (9), 157 (10), 115 (18), 83 (32), 44 (100). Anal. calcd. for $C_{17}H_{20}BrN_3O_2$ (378.26): C 53.98; H 5.33; N 11.11; Found: C 53.96; H 4.99; N 11.16.

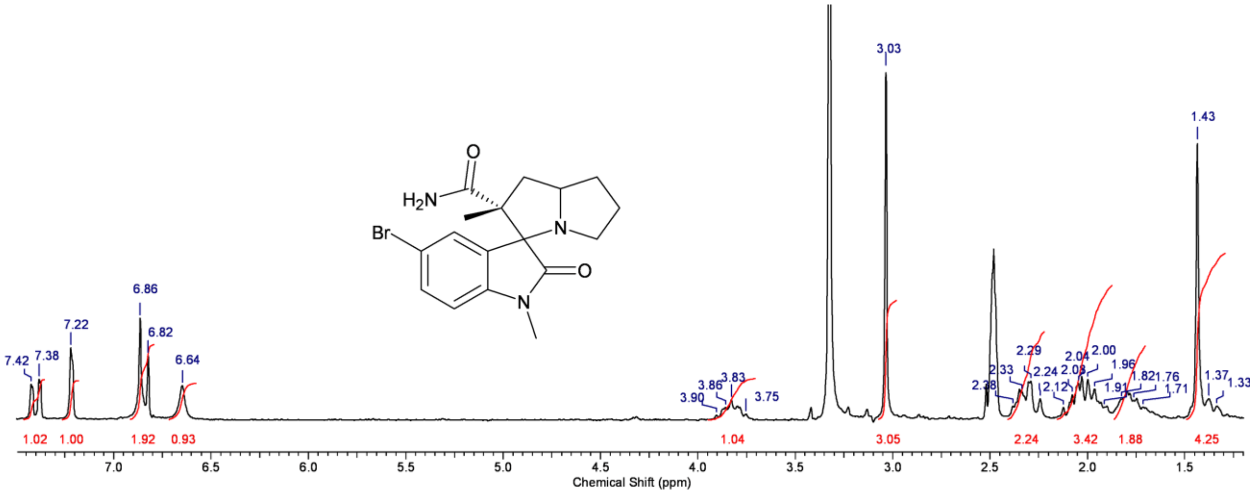

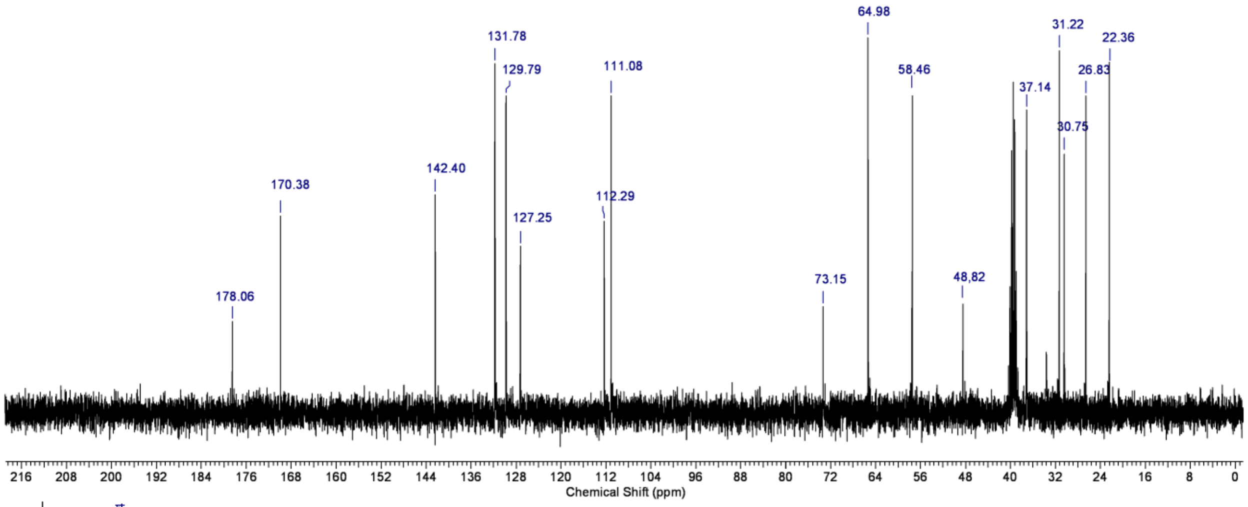

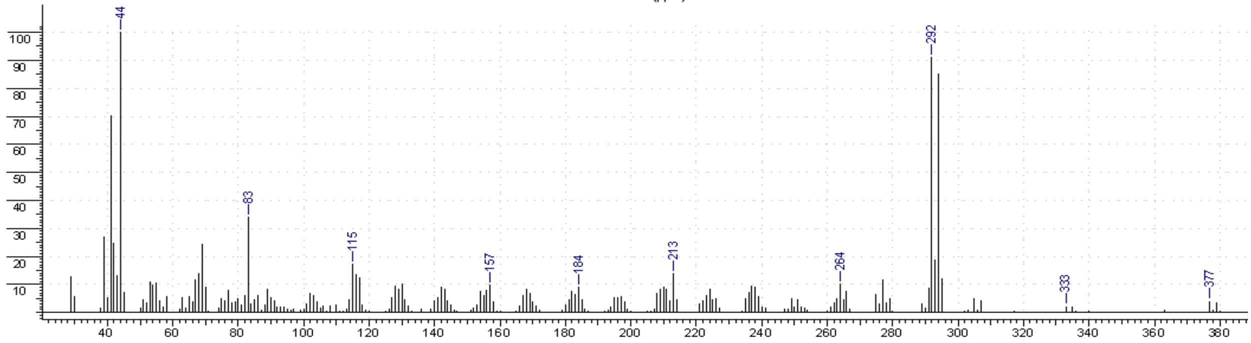

General procedure for the synthesis of spirooxindoles 6a–6h from the three-component reaction of isatins, sarcosine or proline and aroylacrylic acids: A mixture of isatin (1.0 mmol), α-amino acid (1.0 mmol) and aroylacrylic acid (1.0 mmol) in 4.0 mL aqueous methanol (1:3) was heated in an oil bath to reflux temperature for about 20 min or stirred at room temperature from 25 min to 12 hours. The resulting precipitates were collected by filtration and washed with cold methanol to give analytically pure products **6**.

5-Bromo-3'-(3,4-dichlorobenzoyl)-1'-methyl-2-oxo-1,2-dihydrospiro[indole-3,2'-pyrrolidine]-4'-carboxylic acid (6a): colorless solid, 50%, mp 240-242 °C; ^{1}H NMR (200 MHz, DMSO-*d*$_6$) δ: 12.81 (s, 1H, 4'-COOH), 10.65 (s; 1H, 1-NH), 7.63 (d, *J*=8.4 Hz, 1H, 5-CH (3,4-dichlorobenzoyl)), 7.44 (s, 1H, 2-CH (3,4-dichlorobenzoyl)), 7.36 (d, *J*=8.4 Hz, 1H, 6-CH (3,4-dichlorobenzoyl)), 7.21 (d, *J*=8.1 Hz, 1H, 6-CH), 6.96 (s, 1H, 4-CH), 6.44 (d, *J*=8.4 Hz, 1H, 7-CH), 4.51 (d, *J*=9.2 Hz, 1H, 3'-CH), 3.99 (q, *J*=8.4 Hz, 1H, 4'-CH), 3.32-3.12 (m, 2H, 5'-CH_2), 1.96 (s, 3H, 1'-NCH_3); ^{13}C NMR (75 MHz, DMSO-*d*$_6$) δ: 195.09 (CO-benzoyl), 177.42 (2-CO), 173.16 (4'-COOH), 141.26, 136.44, 132.08, 131.70, 130.95, 130.32, 129.02, 128.55, 128.20, 127.13, 113.55, 111.33, 72.27 (C-spiro), 56.54, 54.66, 42.96, 34.40. Anal. calcd. for $C_{20}H_{15}BrCl_2N_2O_4$ (498.15): C 48.22; H 3.04; N 5.62; Found: C 48.17; H 3.10; N 5.67.

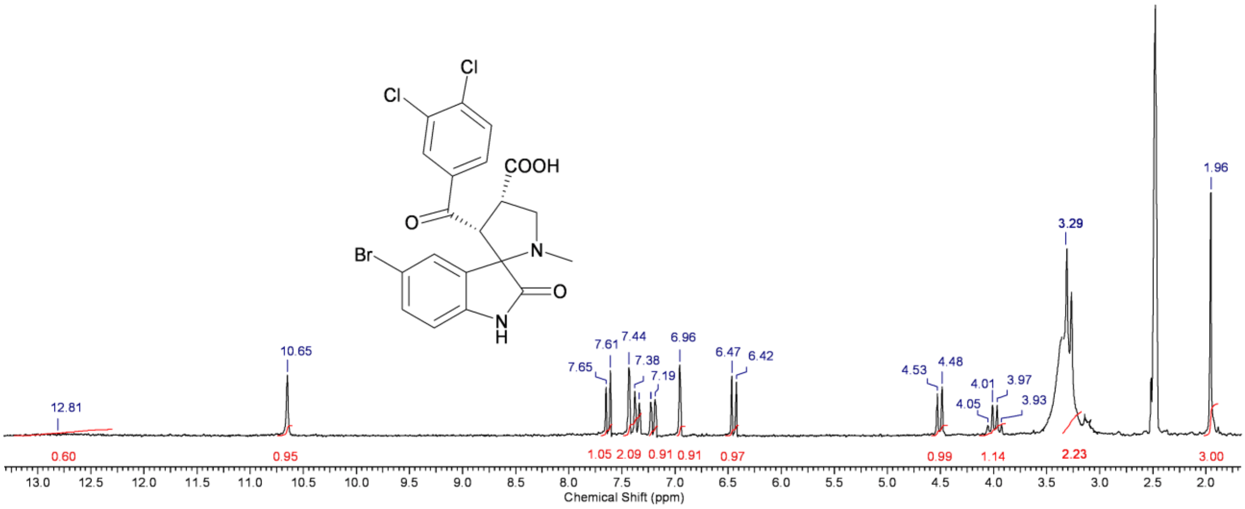

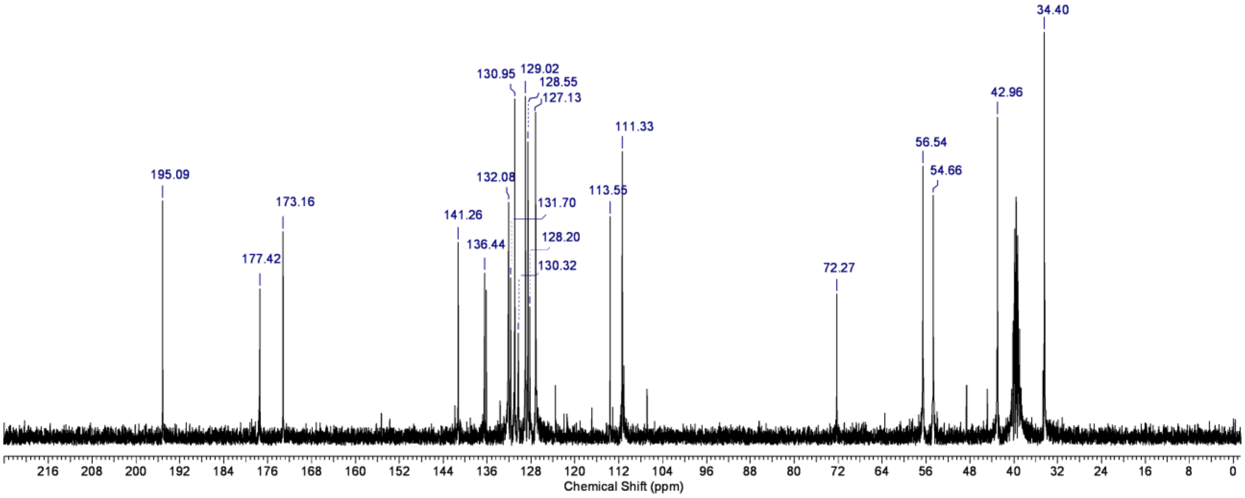

5-Bromo-3'-(4-bromobenzoyl)-1,1'-dimethyl-2-oxo-1,2-dihydrospiro[indole-3,2'-pyrrolidine]-4'-carboxylic acid (6b): colorless solid, 34%, mp 228-229 °C; ^{1}H NMR (200 MHz, DMSO-d_6) δ: 12.55 (s, 1H, 4'-COOH), 10.60 (s; 1H, 1-NH), 7.56 (d, *J*=8.6 Hz, 2H, 3,5-CH (4-bromobenzoyl)), 7.31 (d, 2H, 2,6-CH (4-bromobenzoyl)), 7.18 (dd, *J*=8.2, 2.1 Hz, 1H, 6-CH), 6.96 (d, *J*=1.5 Hz, 1H, 4-CH), 6.44 (d, *J*=8.2 Hz, 1H, 7-CH), 4.52 (d, *J*=8.9 Hz, 1H, 3'-CH), 3.99 (q, *J*=8.6 Hz, 4'-CH), 3.31 (d, *J*=8.6 Hz, 2H, 5'-CH_2), 3.14 (s, 3H, 1-NCH_3), 1.95 (s, 3H, 1'-NCH_3); ^{13}C NMR (75 MHz, DMSO-d_6) δ: 195.09 (CO-benzoyl), 177.39 (2-CO), 173.12 (4'-COOH), 142.38, 135.47, 131.75, 131.48, 129.77, 129.32, 127.24, 127.22, 112.27, 111.09, 72.24 (C-spiro), 56.51, 54.63, 42.93, 34.37, 31.21. Anal. calcd. for $C_{21}H_{18}Br_2N_2O_4$ (522.19): C 48.30; H 3.47; N 5.36; Found: C 48.29; H 3.51; N 5.41.

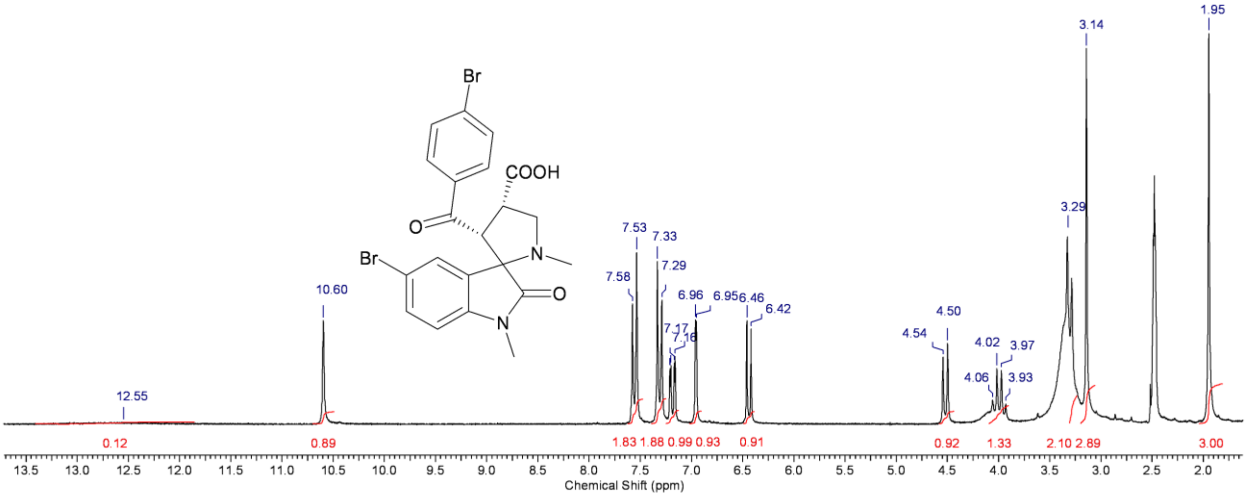

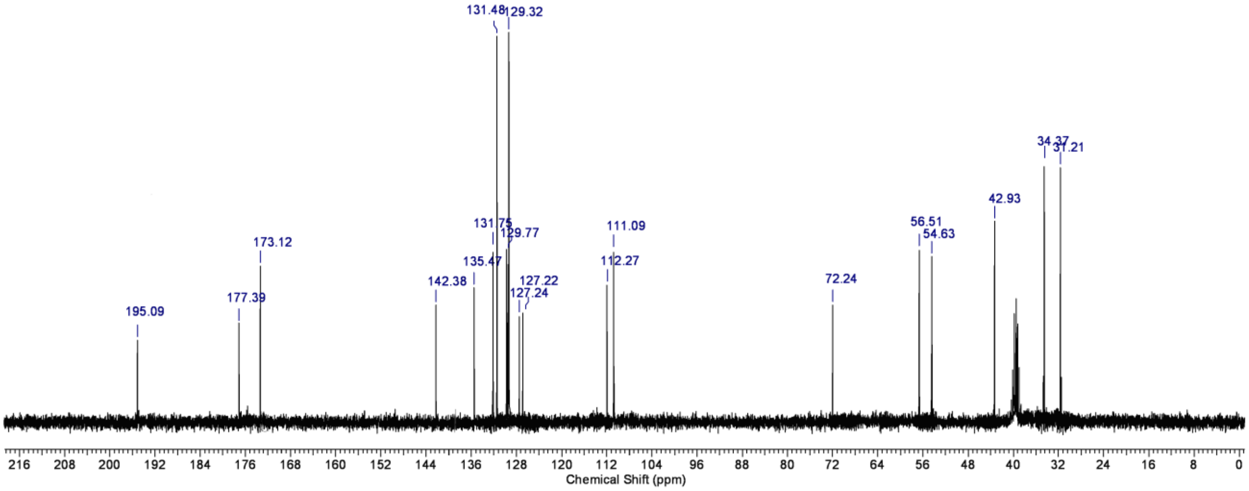

2'-(3,4-Dichlorobenzoyl)-2-oxo-1,1',2,2',5',6',7',7a'-octahydrospiro[indole-3,3'-pyrrolizine]-1'-carboxylic acid (6c): colorless solid, 27% (heating), 67% (room temperature), mp 229-230 °C; ^{1}H NMR (200 MHz, DMSO-d_6) δ: 12.65 (s, 1H, 1’-COOH), 10.25 (s; 1H, 1-NH), 7.61 (d, *J*=8.1 Hz, 1H, 5-CH (3,4-dichlorobenzoyl)), 7.30 (s, 1H, 2-CH (3,4-dichlorobenzoyl)), 7.22 (d, *J*=8.4 Hz, 1H, 6-CH (3,4-dichlorobenzoyl)), 7.14-6.99 (m, 2H, 4,5-CH), 6.68 (t, *J*=7.3 Hz, 1H, 6-CH), 6.48 (d, *J*=7.6 Hz, 1H, 7-CH), 4.67 (d, *J*=11.4 Hz, 1H, 2’-CH), 4.02-3.66 (m, 1H, 7a’-CH), 3.68-3.35 (m, 1H, 1’-CH), 2.42-2.17 (m, 2H, 5’-CH_2), 2.09-1.61 (m, 4H, 7’,6’-CH_2); ^{13}C NMR (75 MHz, DMSO-d_6) δ: 195.29 (CO-benzoyl), 178.81 (2-CO), 172.80 (1’-COOH), 141.83, 136.55, 135.92, 131.48, 130.80, 129.55, 129.05, 127.24, 127.02, 124.17, 121.36, 109.71, 71.56 (C-spiro), 66.98, 59.94, 50.58, 47.11, 30.76, 26.86; MS (m/z) (%):444 (M^+, 2), 371 (4), 355 (4), 296 (3), 243 (17), 200 (100), 173 (63), 145 (58), 109

(26), 41 (23). Anal. calcd. for $C_{22}H_{18}Cl_2N_2O_4$ (445.30): C 59.34; H 4.07; N 6.29; Found: C 59.39; H 4.11; N 6.27.

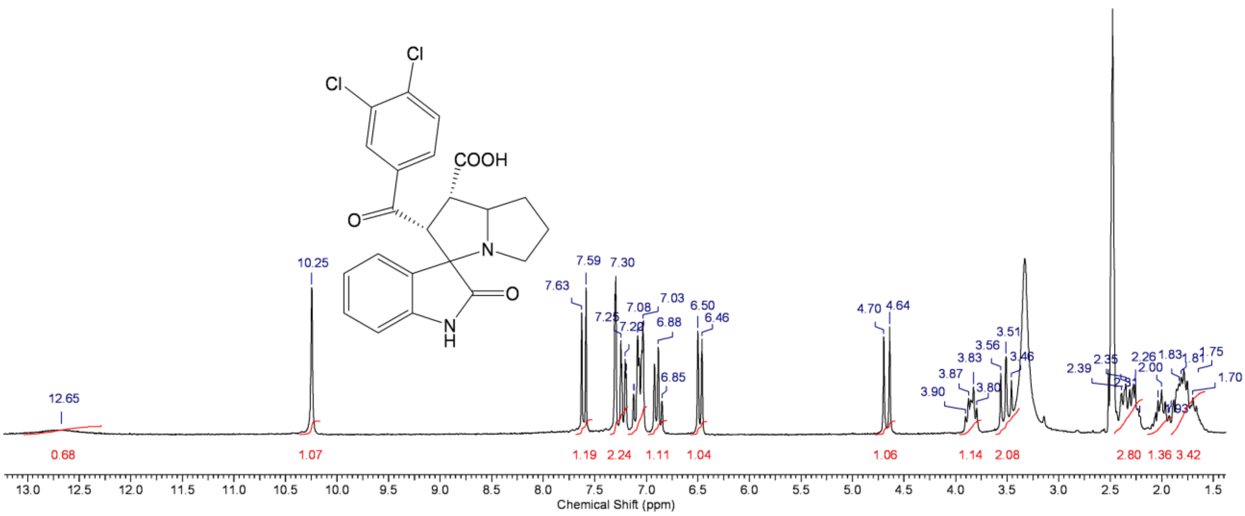

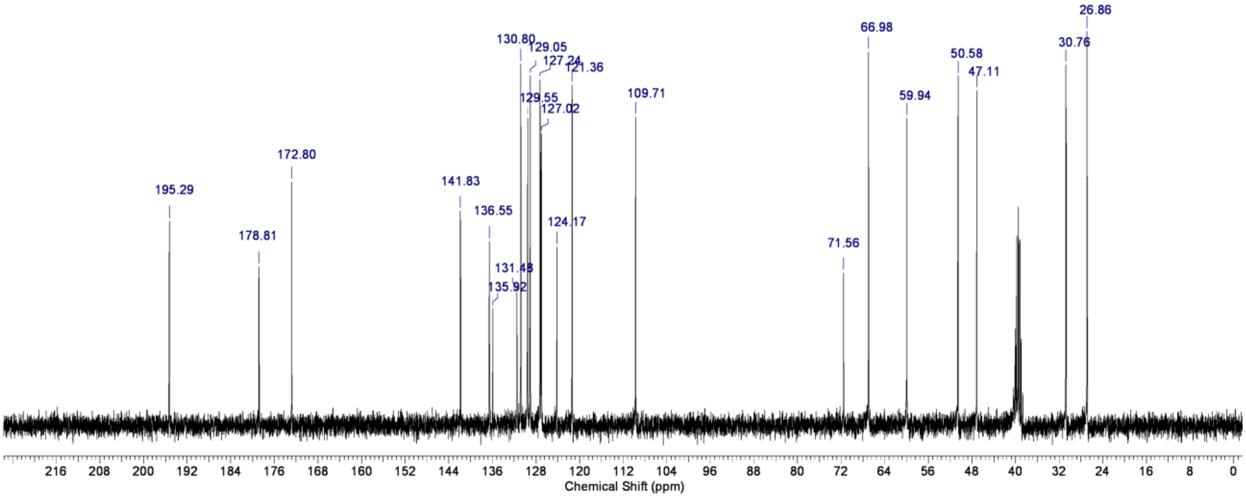

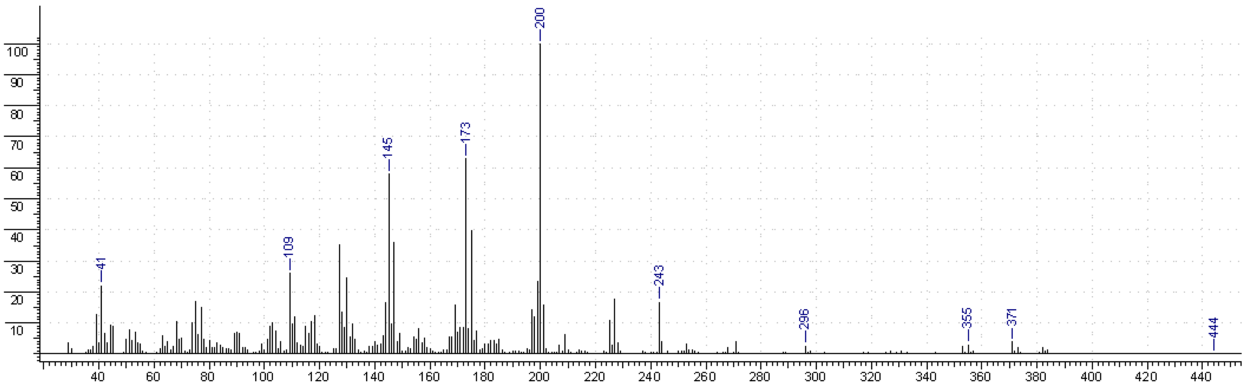

NOE

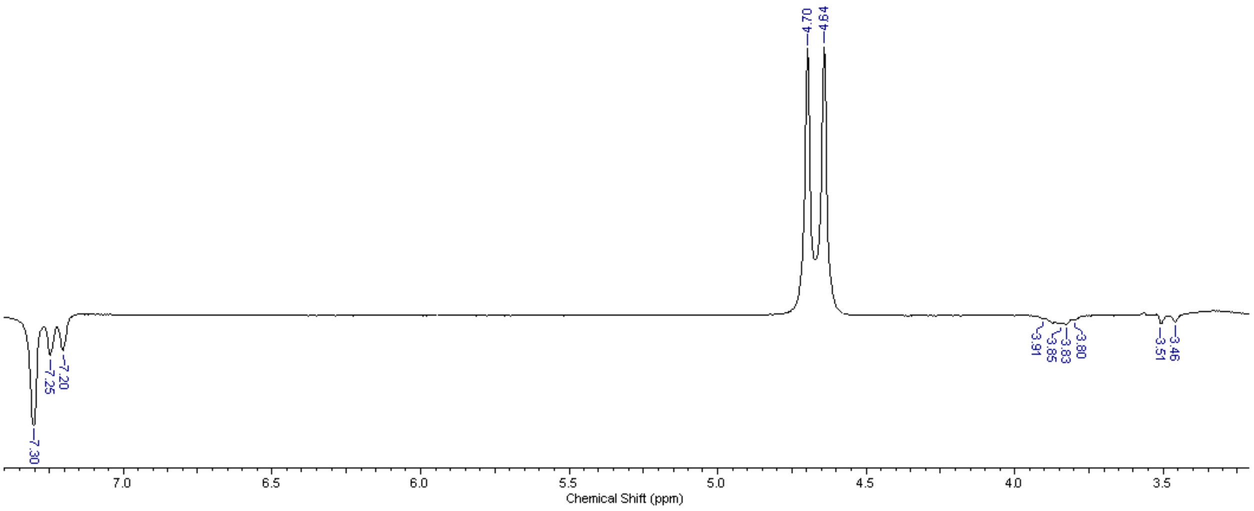

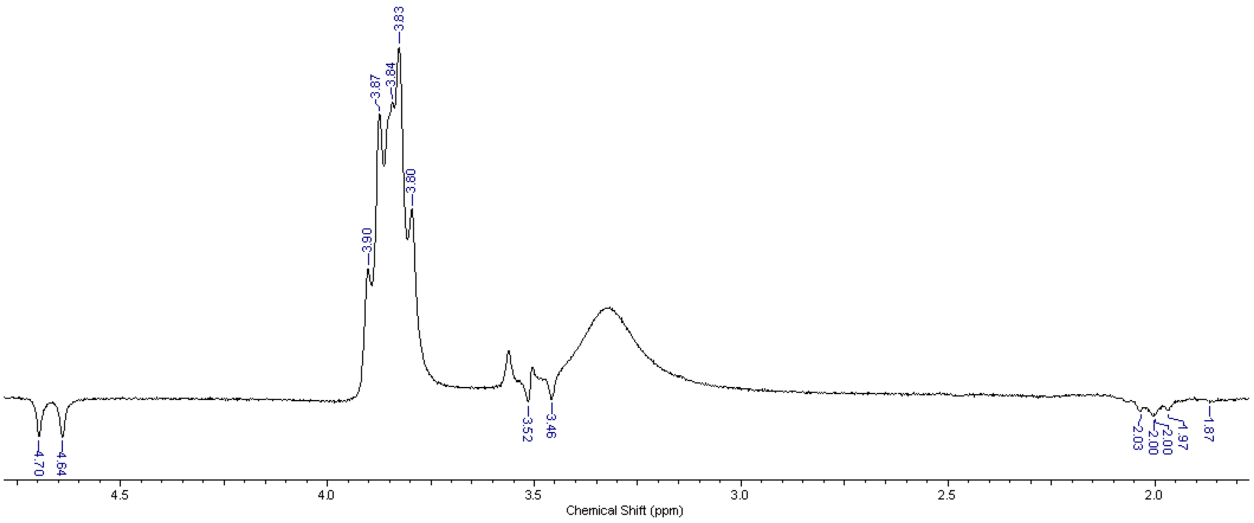

2'-(3,4-Dichlorobenzoyl)-1,5-dimethyl-2-oxo-1,1',2,2',5',6',7',7a'-octahydrospiro[indole-3,3'-pyrrolizine]-1'-carboxylic acid (6d): colorless solid, 30%, mp 229-230 °C; ^{1}H NMR (200 MHz, DMSO-d_6) δ: 12.58 (s, 1H, 1’-COOH), 7.58 (d, *J*=8.2 Hz, 1H, 5-CH (3,4-dichlorobenzoyl)), 7.29-6.74 (m, 4H, 2,6-CH (3,4-dichlorobenzoyl), 4,6-CH), 6.51 (d, *J*=7.9 Hz, 1H, 7-CH), 4.63 (d, *J*=11.3 Hz, 1H, 2’-CH), 3.97-3.64 (m, 1H, 7a’-CH), 3.59-3.27 (m, 1H, 1’-CH), 2.69 (s, 3H, 1-NCH_3), 2.35-2.04 (m, 4H, 1’-NCH_3, 5’-CH_2), 2.10-1.52 (m, 5H, 5’,7’,6’-CH_2); ^{13}C NMR (75 MHz, DMSO-d_6) δ: 195.29 (CO-benzoyl), 178.81 (2-CO), 173.12 (1’-COOH), 140.23, 136.55, 135.92, 131.48, 130.80, 129.55, 129.05, 127.24, 124.62, 124.17, 114.15, 113.60, 71.56 (C-spiro), 66.98, 59.94, 50.58, 47.11, 31.22, 30.76, 27.66, 26.86. Anal. calcd. for $C_{24}H_{22}Cl_2N_2O_4$ (473.35):C 60.90; H 4.68; N 5.92; Found: C 60.93; H 4.79; N 5.98.

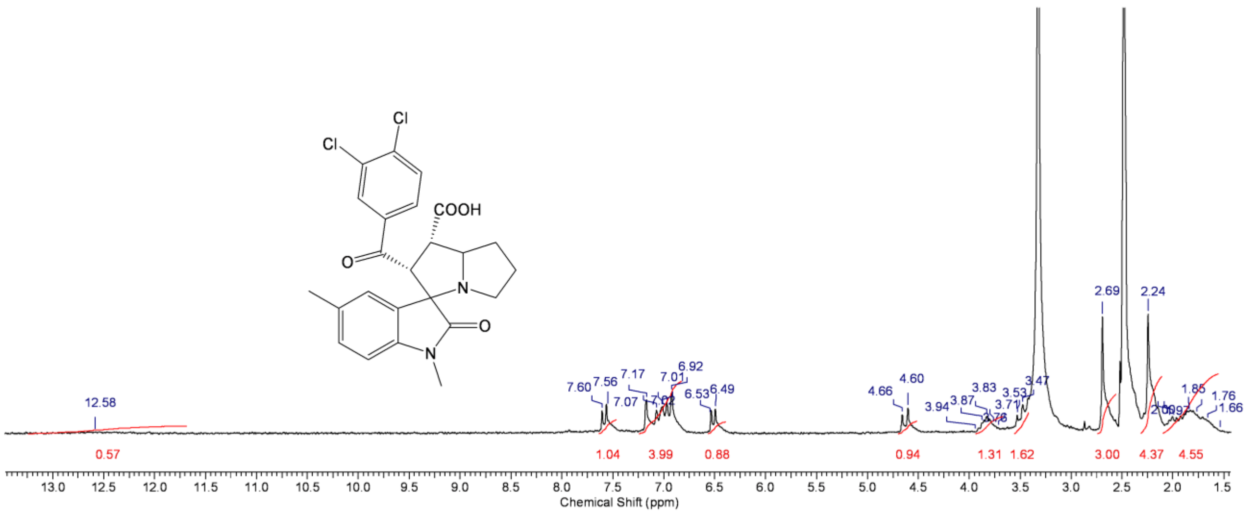

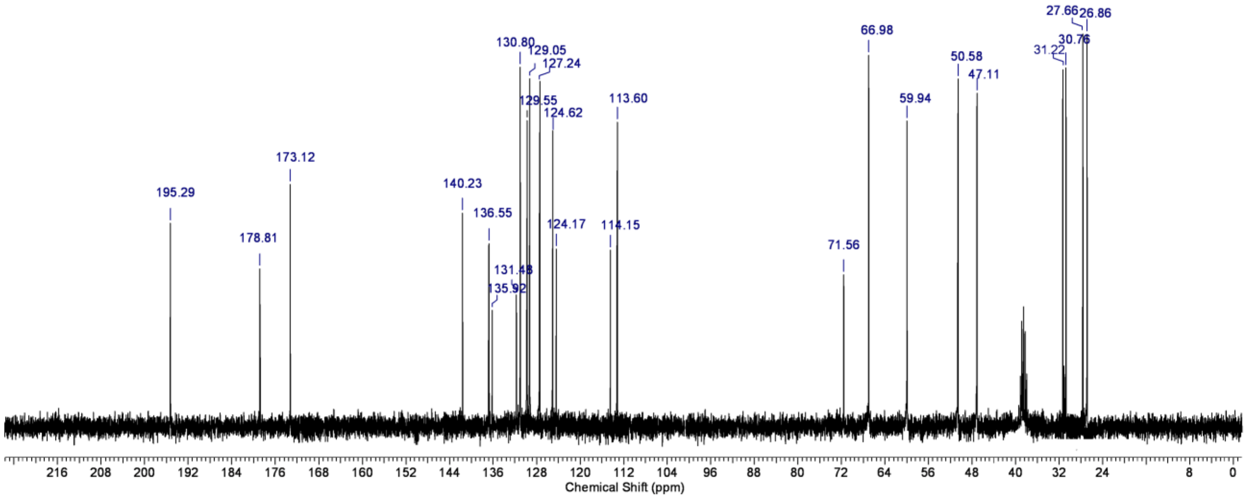

2'-(4-Bromobenzoyl)-2-oxo-1,1',2,2',5',6',7',7a'-octahydrospiro[indole-3,3'-pyrrolizine]-1'-carboxylic acid (6e): colorless solid, 15% (heating), 57% (room temperature), mp 231-232 °C; ^{1}H NMR (200 MHz, DMSO-d_6) δ: 12.57 (s, 1H, 1'-COOH), 10.20 (s, 1H, 1-NH), 7.53 (d, J=8.2 Hz, 2H, 3,5-CH (4-bromobenzoyl)), 7.21 (d, J=8.5 Hz, 2H, 2,6-CH (4-bromobenzoyl), 7.12-6.98 (m, 2H, 4,5-CH), 6.87 (t, J=7.5 Hz, 1H, 6-CH), 6.48 (d, J=7.6 Hz, 1H, 7-CH), 4.70 (d, J=11.0 Hz, 1H, 2'-CH), 3.93-3.77 (m, 1H, 7a'-CH), 3.61-3.46 (m, 1H, 1'-CH), 2.42-2.18 (m, 2H, 5'-CH_2), 2.10-1.59 (m, 4H, 7',6'-CH_2); ^{13}C NMR (75 MHz, DMSO-d_6) δ: 196.23 (CO-benzoyl), 178.86 (2-CO), 172.94 (1'-COOH), 141.81, 135.49, 131.48, 129.47, 129.32, 127.24, 127.05, 124.30, 121.24, 109.69, 71.57 (C-spiro), 66.91, 59.44, 50.77, 47.10, 30.77, 26.86. Anal. calcd. for $C_{22}H_{19}BrN_2O_4$ (455.30); C 58.04; H 4.21; N 6.15; Found: C 57.96; H 4.27; N 6.16.

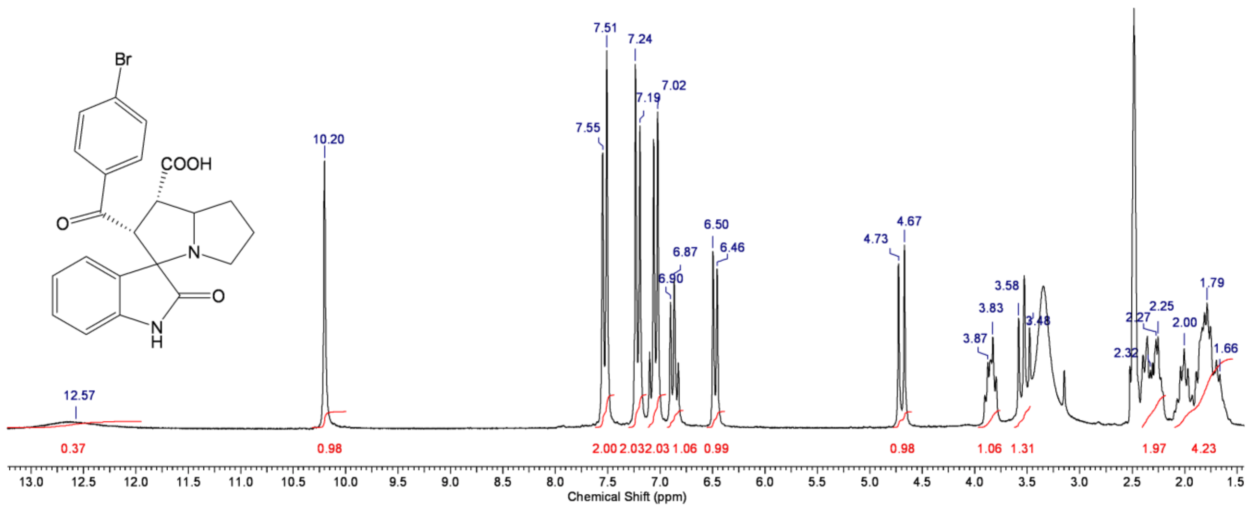

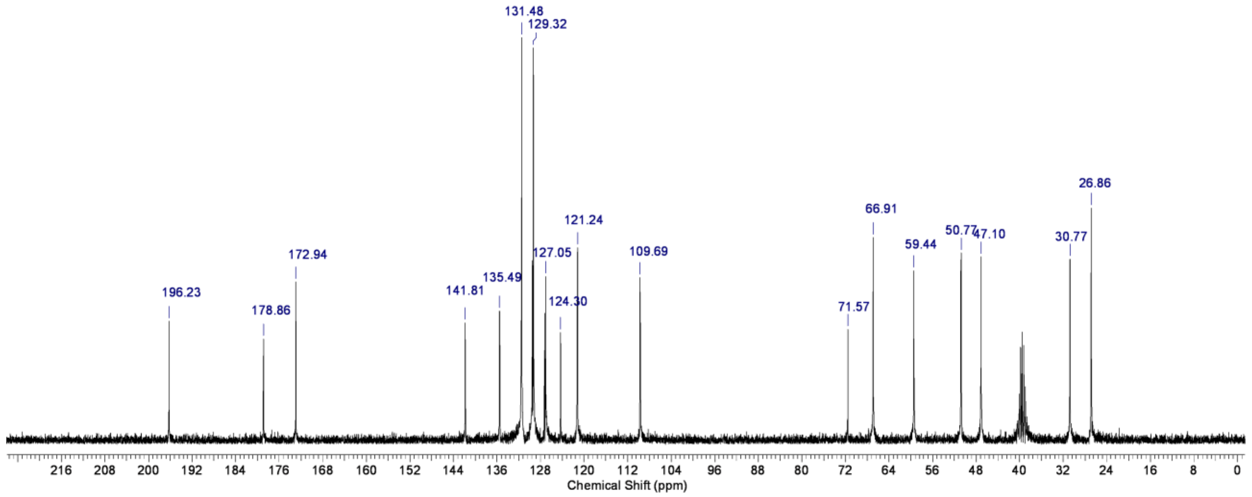

2'-(4-Nitrobenzoyl)-2-oxo-1,1',2,2',5',6',7',7a'-octahydrospiro[indole-3,3'-pyrrolizine]-1'-carboxylic acid (6f): colorless solid, 74% (heating), 76% (room temperature), mp 248-249 °C; ^{1}H NMR (200 MHz, DMSO-d_6) δ: 12.75 (s, 1H, 1'-COOH), 10.09 (s, 1H, 1-NH), 8.12 (d, *J*=8.6 Hz, 2H, 3,5-CH (4-nitrobenzoyl)), 7.39 (d, *J*=8.6 Hz, 2H, 2,6-CH (4-nitrobenzoyl), 7.17-6.99 (m, 2H, 4,5-CH), 6.89 (t, *J*=7.2 Hz, 1H, 6-CH), 6.41 (d, *J*=7.3 Hz, 1H, 7-CH), 4.76 (d, *J*=11.3 Hz, 1H, 2'-CH), 3.97-3.76 (m, 1H, 7a'-CH), 3.60-3.43 (m, 1H, 1'-CH), 2.42-2.17 (m, 2H, 5'-CH_2), 2.12-1.58 (m, 4H, 7',6'-CH_2); ^{13}C NMR (75 MHz, DMSO-d_6) δ: 196.22 (CO-benzoyl), 178.86 (2-CO), 172.94 (1'-COOH), 147.32, 141.97, 141.81, 131.08, 129.44, 127.05, 124.30, 122.25, 121.24, 109.69, 71.57 (C-spiro), 66.91, 59.45, 50.77, 47.12, 30.78, 26.86. Anal. calcd. for $C_{22}H_{19}N_3O_6$ (421.40): C 62.70; H 4.54; N 9.97; Found: C 62.66; H 4.59; N 10.01.

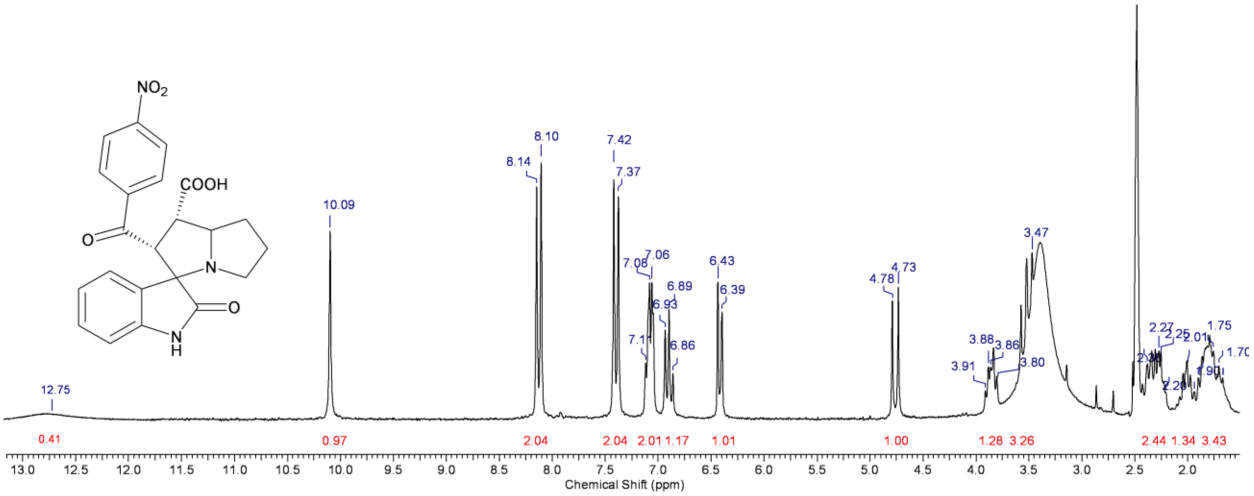

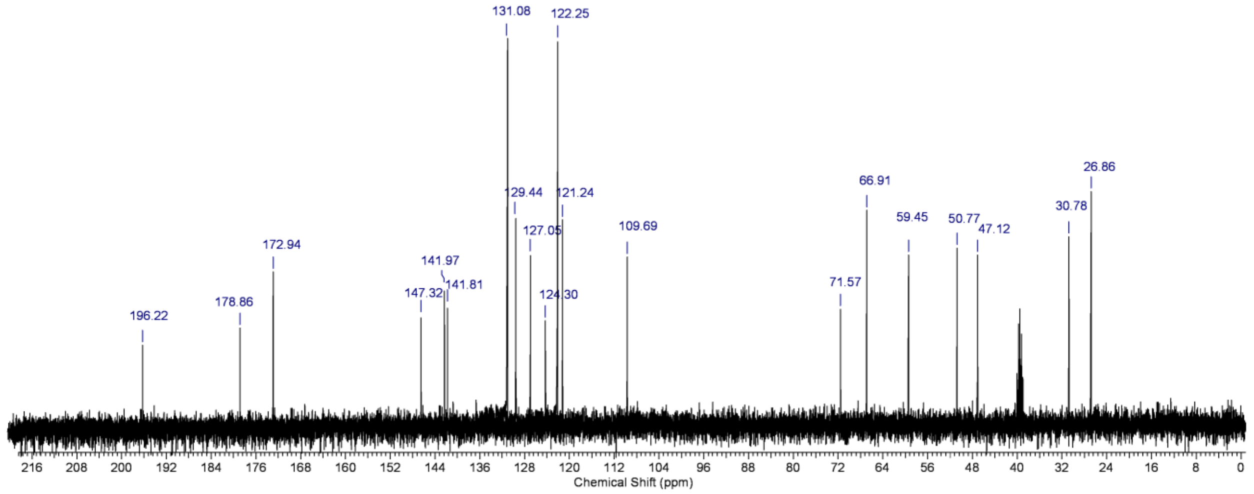

5-Methyl-2'-(4-nitrobenzoyl)-2-oxo-1,1',2,2',5',6',7',7a'-octahydrospiro[indole-3,3'-pyrrolizine]-1'-carboxylic acid (6g): colorless solid, 30% (heating), 74% (room temperature), mp 231-232 °C; ^{1}H NMR (200 MHz, DMSO-d_6) δ: 12.80 (s, 1H, 1’-COOH), 9.98 (s, 1H, 1-NH), 8.13 (d, *J*=8.6 Hz, 2H, 3,5-CH (4-nitrobenzoyl)), 7.41 (d, *J*=8.6 Hz, 2H, 2,6-CH (4-nitrobenzoyl), 6.99-6.82 (m, 2H, 4,6-CH), 6.30 (d, *J*=7.9 Hz, 1H, 7-CH), 4.75 (d, *J*=11.3 Hz, 1H, 2’-CH), 3.98-3.76 (m, 1H, 7a’-CH), 3.63-3.44 (m, 1H, 1’-CH), 2.45-2.16 (m, 5H, 5’-CH_2, 5-CH_3), 2.13-1.55 (m, 4H, 7’,6’-CH_2); ^{13}C NMR (75 MHz, DMSO-d_6) δ: 195.31 (CO-benzoyl), 178.80 (2-CO), 172.94 (1’-COOH), 147.34, 146.50, 141.98, 138.68, 131.90, 131.10, 124.69, 122.25, 117.68, 112.01, 71.56 (C-spiro), 66.91, 59.44, 50.78, 47.12, 30.76, 26.88, 21.60. Anal. calcd. for $C_{23}H_{21}N_3O_6$ (435.43):C 63.44; H 4.86; N 9.65; Found: C 63.46; H 4.89; N 9.69.

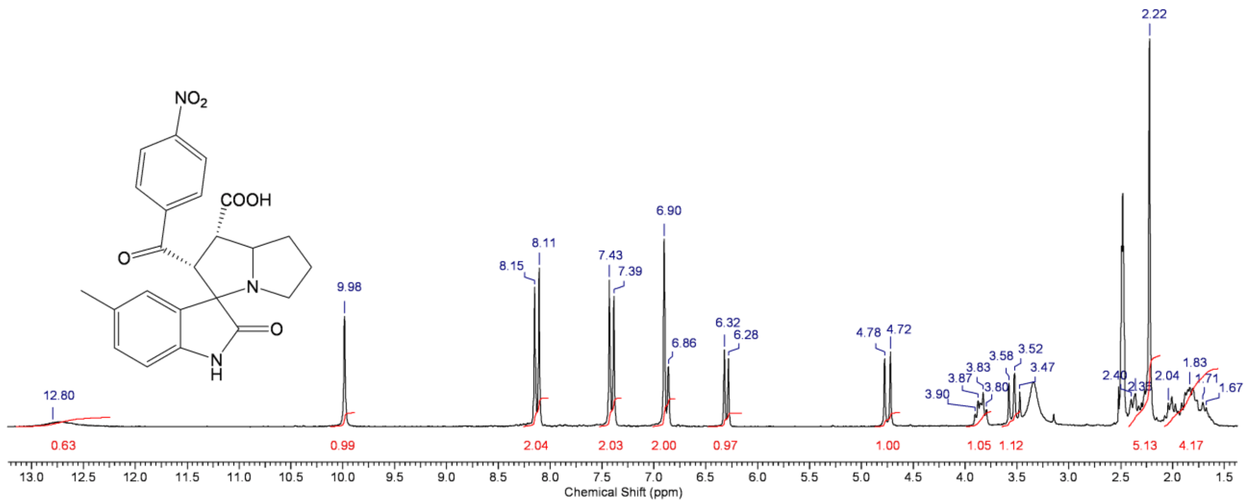

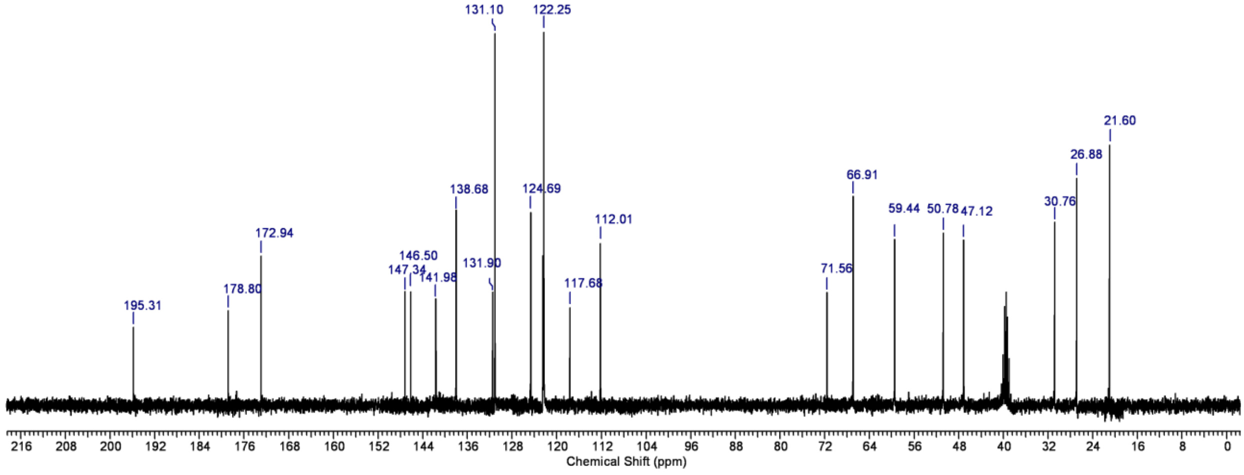

5-Bromo-2'-(4-nitrobenzoyl)-2-oxo-1,1',2,2',5',6',7',7a'-octahydrospiro[indole-3,3'-pyrrolizine]-1'-carboxylic acid (6h): colorless solid, 50% (heating), 76% (room temperature), mp 239-240 °C; ^{1}H NMR (200 MHz, DMSO-d_6) δ: 12.74 (s, 1H, 1’-COOH), 10.26 (s, 1H, 1-NH), 8.17 (d, *J*=8.6 Hz, 2H, 3,5-CH (4-nitrobenzoyl)), 7.47 (d, *J*=8.6 Hz, 2H, 2,6-CH (4-nitrobenzoyl), 7.35-7.22 (m, 2H, 4,6-CH), 6.40 (d, *J*=8.9 Hz, 1H, 7-CH), 4.78 (d, *J*=11.3 Hz, 1H, 2’-CH), 3.94-3.75 (m, 1H, 7a’-CH), 3.67-3.50 (m, 1H, 1’-CH), 2.44-2.19 (m, 2H, 5’-CH_2), 2.09-1.59 (m, 4H, 7’,6’-CH_2); ^{13}C NMR (75 MHz, DMSO-d_6) δ: 196.21 (CO-benzoyl), 177.40 (2-CO), 172.94 (1’-COOH), 147.34, 142.38, 141.98, 131.75, 131.10, 129.77, 127.23, 122.25, 112.28, 111.07, 71.56 (C-spiro), 66.91, 59.44, 50.78, 47.12, 30.76, 26.88. Anal. calcd. for $C_{22}H_{18}BrN_3O_6$ (500.30):C 52.82; H 3.63; N 8.40; Found: C 52.92; H 3.79; N 8.45.

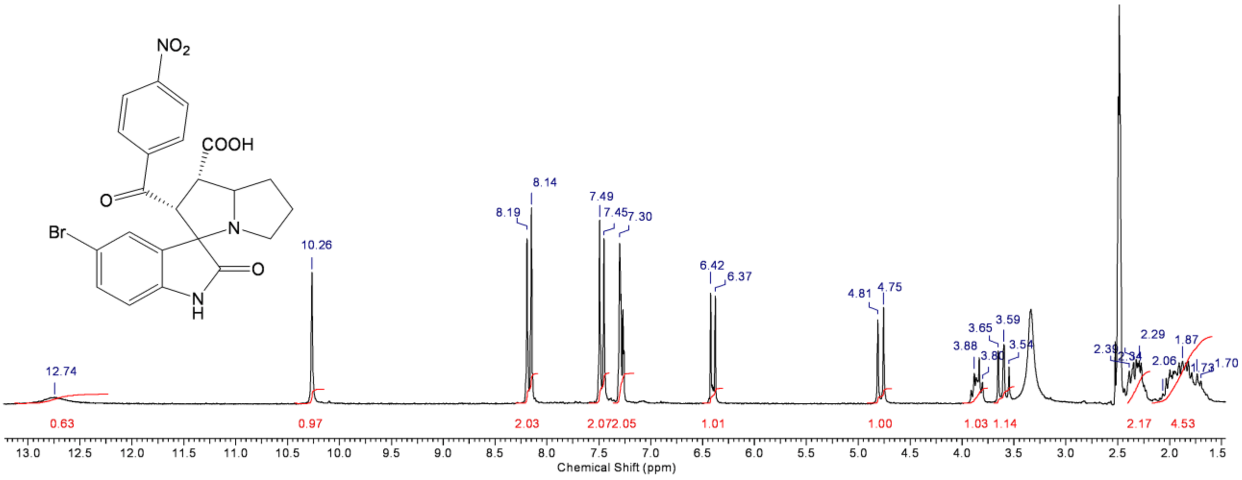

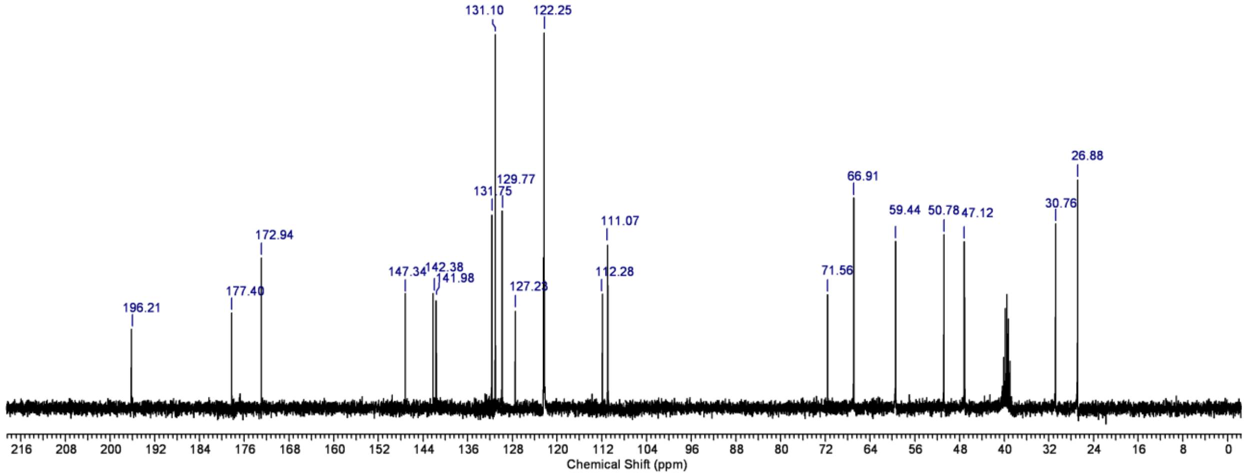

General procedure for synthesis of compounds 7a–7c from the three-component reaction of isatins, proline and aroylacrylic acids: A mixture of isatin (1.0 mmol), proline (1.0 mmol) and aroylacrylic acid (1.0 mmol) in 4.0 mL aqueous ethanol (1:3) was heated in an oil bath to reflux temperature for 15 min. The resulting precipitate was collected by filtration and washed with cold ethanol to give analytically pure products **7**.

5-Bromo-3-[5-(3,4-dichlorophenyl)-2,3-dihydro-1*H*-pyrrolizin-6-yl]-1,3-dihydro-2*H*-indol-2-one (7a): orange powder, 22%, mp 215-216 °C. MS (m/z) (%): 462 (M^+, 52), 435 (45), 405 (8), 353 (12), 317 (18), 289 (52), 208 (30), 173 (33), 127 (46), 75 (30), 41 (100). Anal. calcd. for $C_{21}H_{15}BrCl_2N_2O$ (462.17): C 54.57; H 3.27; N 6.06; Found: C 54.48; H 3.19; N 6.10.

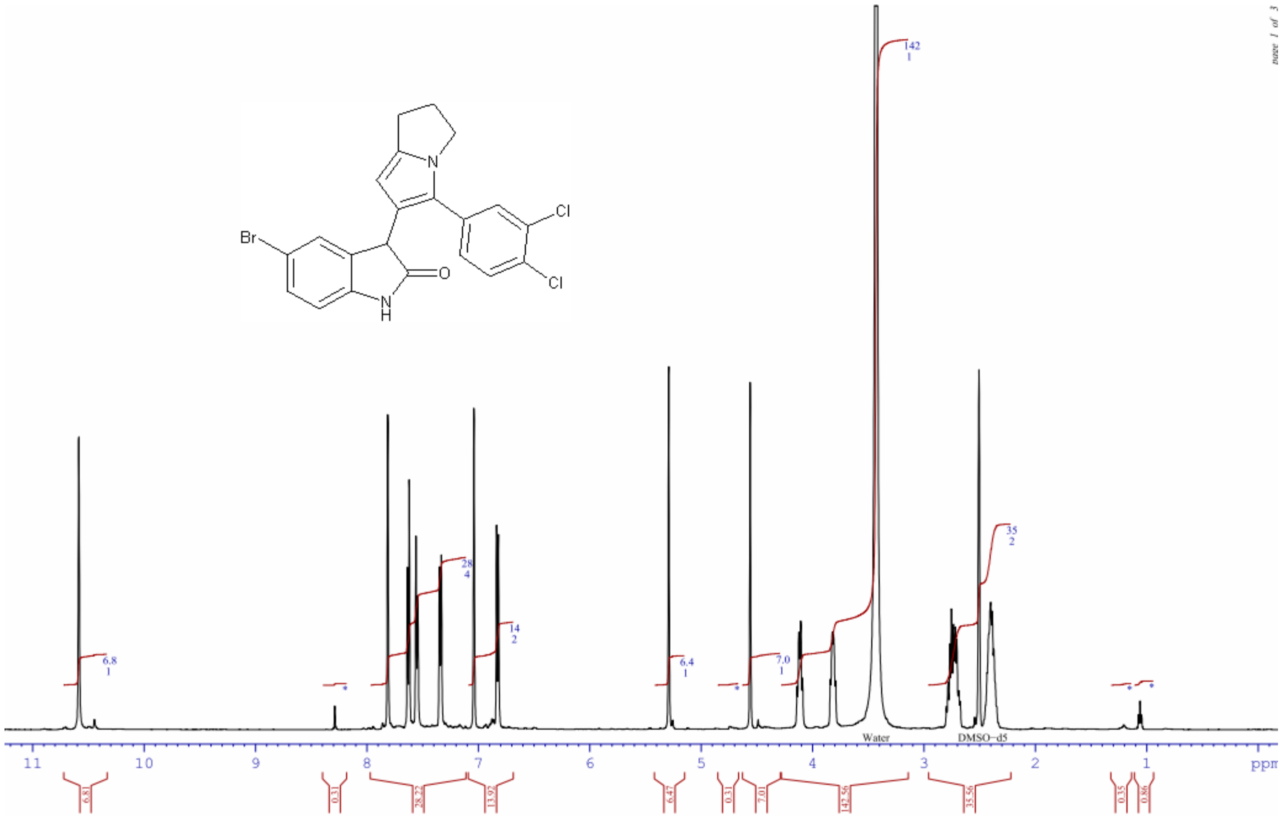

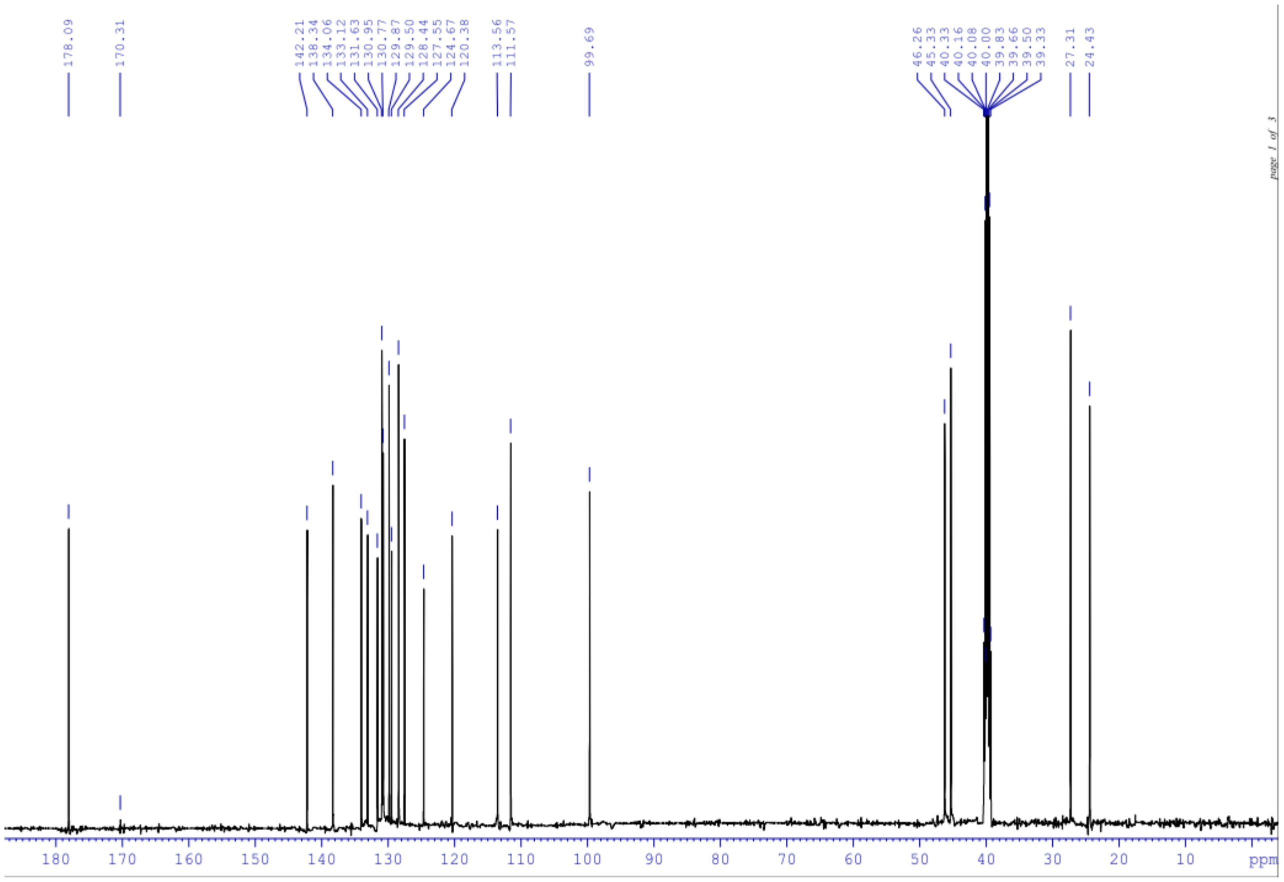

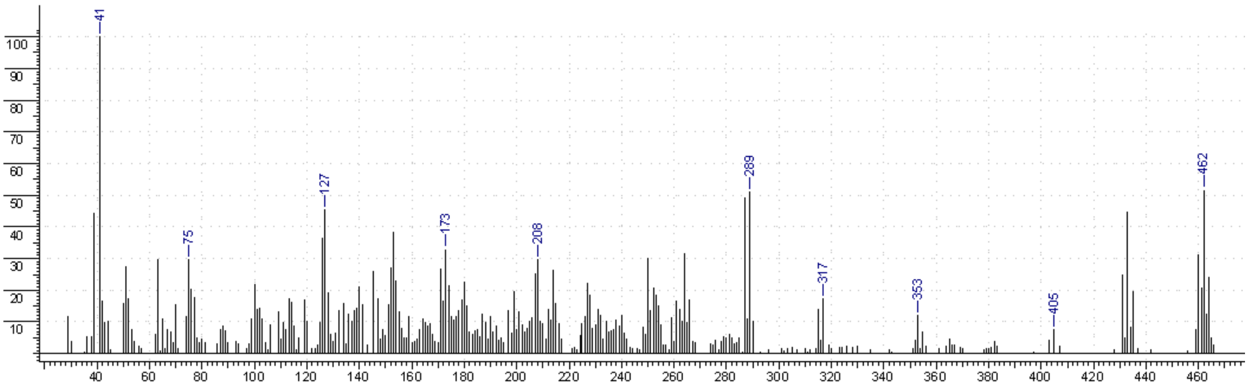
41
75
127
173
208
289
317
353
405
462
100
90
80
70
60
50
40
30
20
10
40 60 80 100 120 140 160 180 200 220 240 260 280 300 320 340 360 380 400 420 440 460

COSY

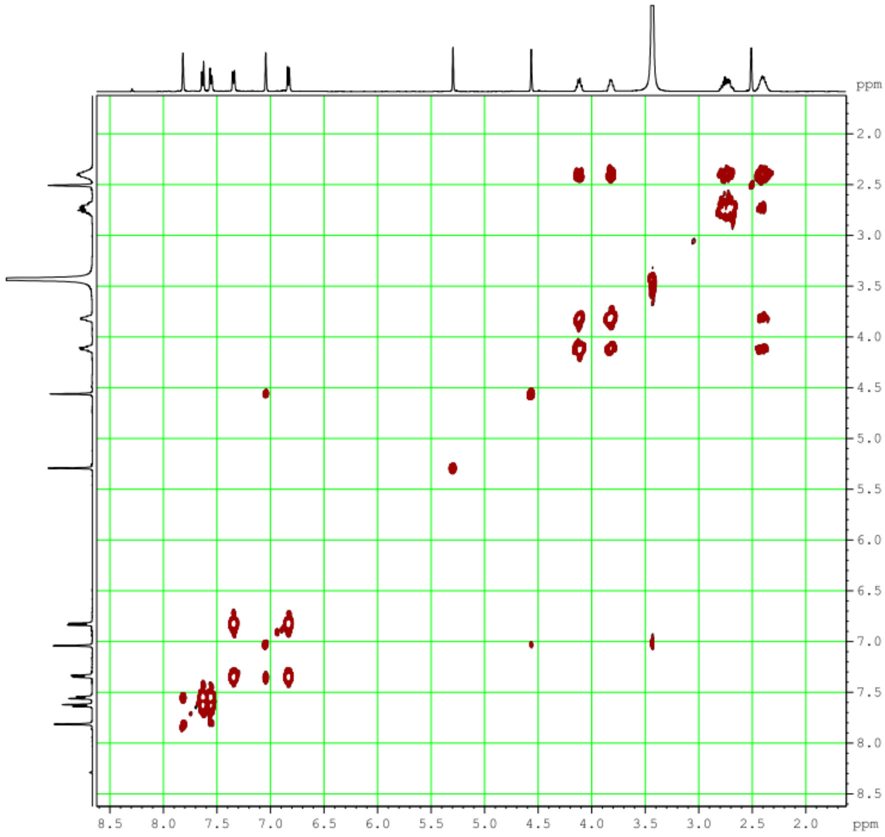
ppm
2.0
2.5
3.0
3.5
4.0
4.5
5.0
5.5
6.0
6.5
7.0
7.5
8.0
8.5
8.5 8.0 7.5 7.0 6.5 6.0 5.5 5.0 4.5 4.0 3.5 3.0 2.5 2.0
ppm

NOESY

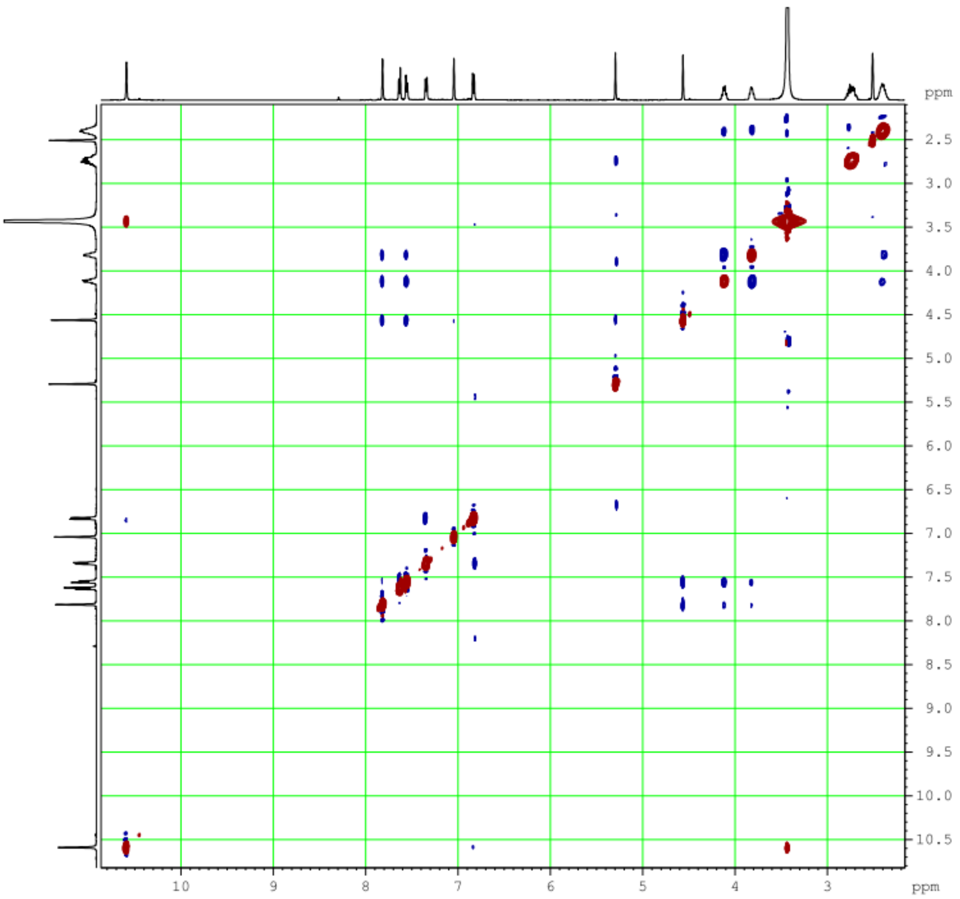

ppm
2.5
3.0
3.5
4.0
4.5
5.0
5.5
6.0
6.5
7.0
7.5
8.0
8.5
9.0
9.5
10.0
10.5
10
9
8
7
6
5
4
3
ppm

HSQC

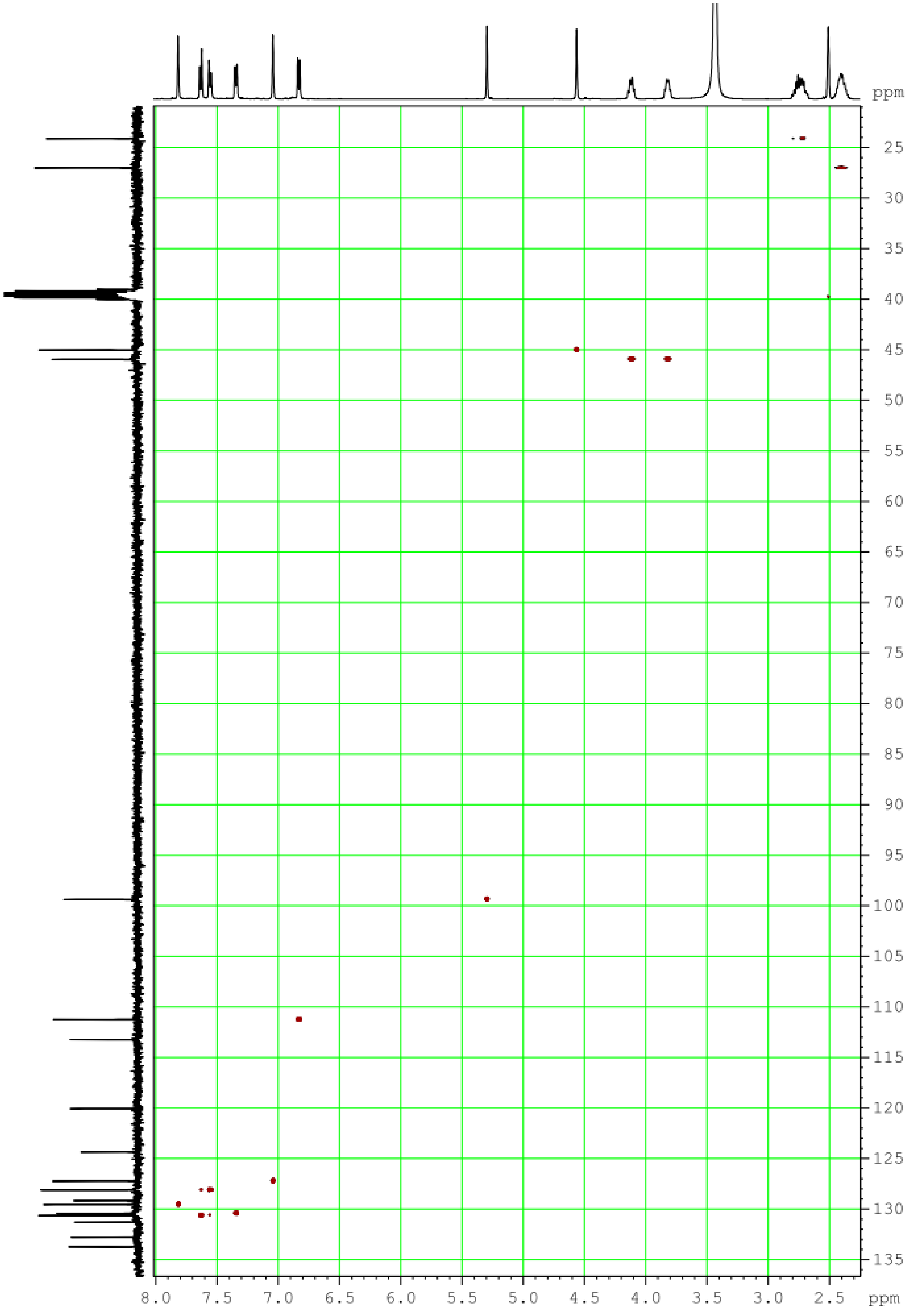
ppm
25
30
35
40
45
50
55
60
65
70
75
80
85
90
95
100
105
110
115
120
125
130
135
8.0
7.5
7.0
6.5
6.0
5.5
5.0
4.5
4.0
3.5
3.0
2.5
ppm

HMBC

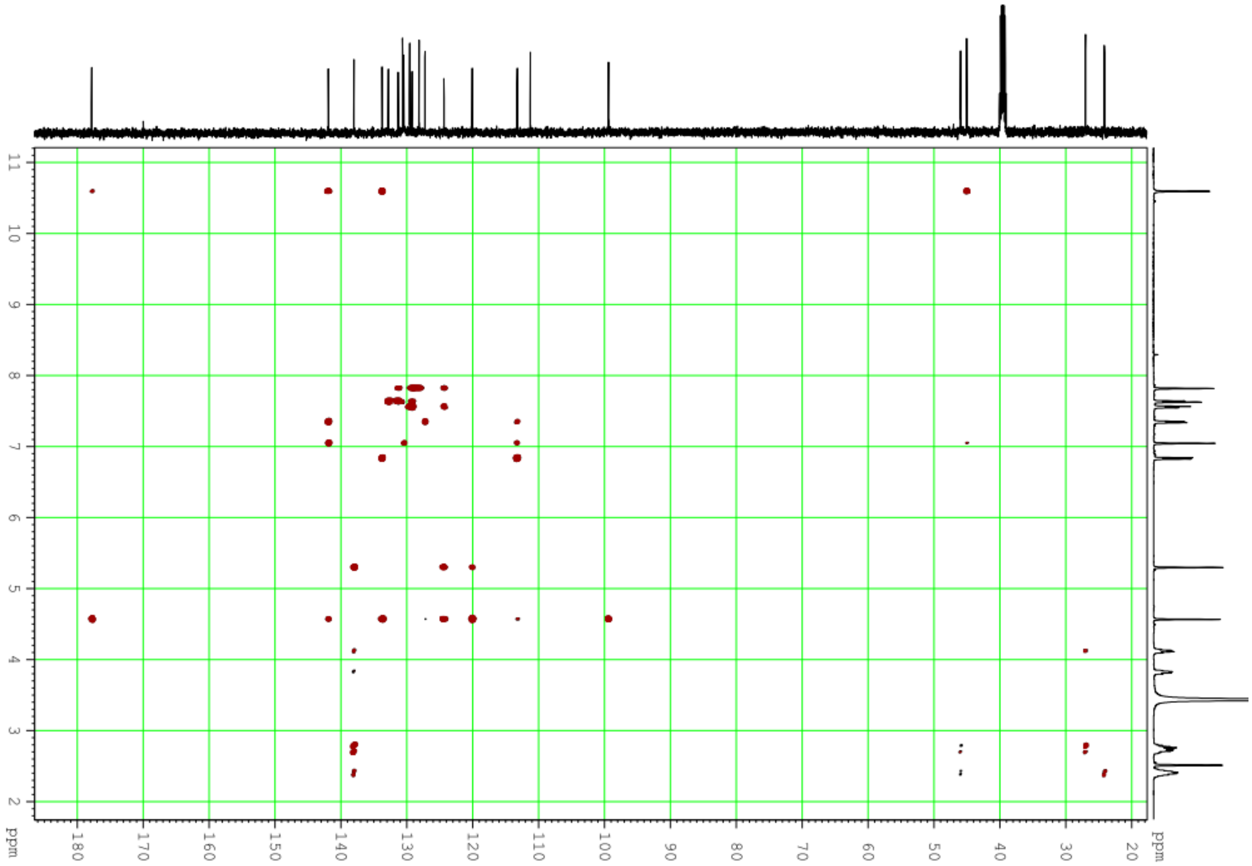

5-Bromo-3-[5-(4-nitrophenyl)-2,3-dihydro-1*H*-pyrrolizin-6-yl]-1,3-dihydro-2*H*-indol-2-one (7b): brown powder, 23 %, mp 209-210 °C; ^{1}H NMR (200 MHz, DMSO-d_6) δ: 10.61 (s, 1H, 1-NH), 8.23 (d, *J*=8.9 Hz, 2H, 3,5-CH (4-nitrobenzoyl)), 7.86 (d, *J*=8.9 Hz, 2H, 2,6-CH (4-nitrobenzoyl), 7.35 (dd, *J*=8.2 Hz, *J*=2.1 Hz,1H, 6-CH (oxindol)), 7.05 (s, 1H, 4-CH (oxindol)), 6.82 (d, *J*=8.2 Hz, 1H, 7-CH (oxindol)), 5.31 (s, 1H, 7-CH), 4.65 (s, 1H, 3-CH), 4.31-3.79 (m, 2H, 3-CH_2), 2.86-2.64 (m, 2H, 1-CH_2), 2.46-2.30 (m, 2H, 2-CH_2); ^{13}C NMR (75 MHz, DMSO-d_6) δ: 177.40 (2-CO), 147.34, 142.38,141.98, 138.34, 131.75, 131.10, 129.77, 127.23, 122.25, 120.33, 120.18, 111.07, 99.23, 46.26, 45.33, 27.31, 24.43. Anal. calcd. for $C_{21}H_{16}BrN_3O_3$ (438.27):C 57.55; H 3.68; N 9.59; Found: C 57.60; H 3.71; N 9.61.

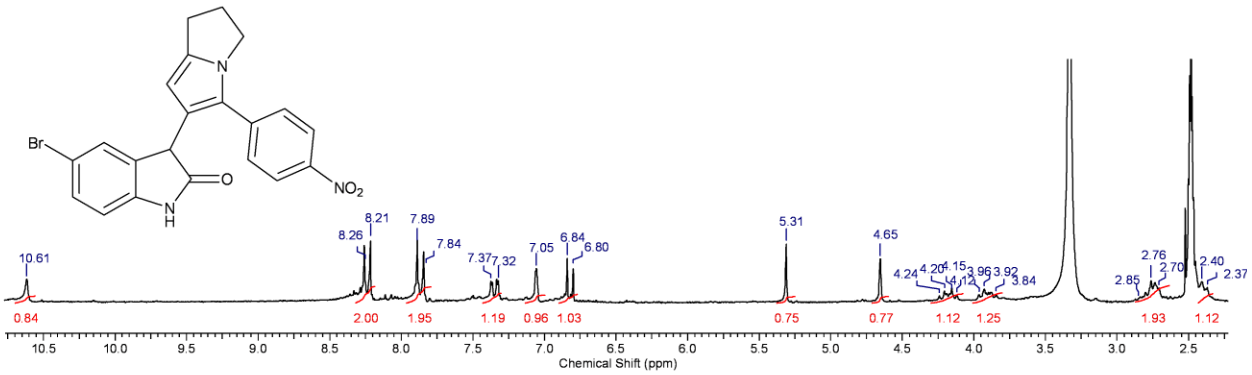

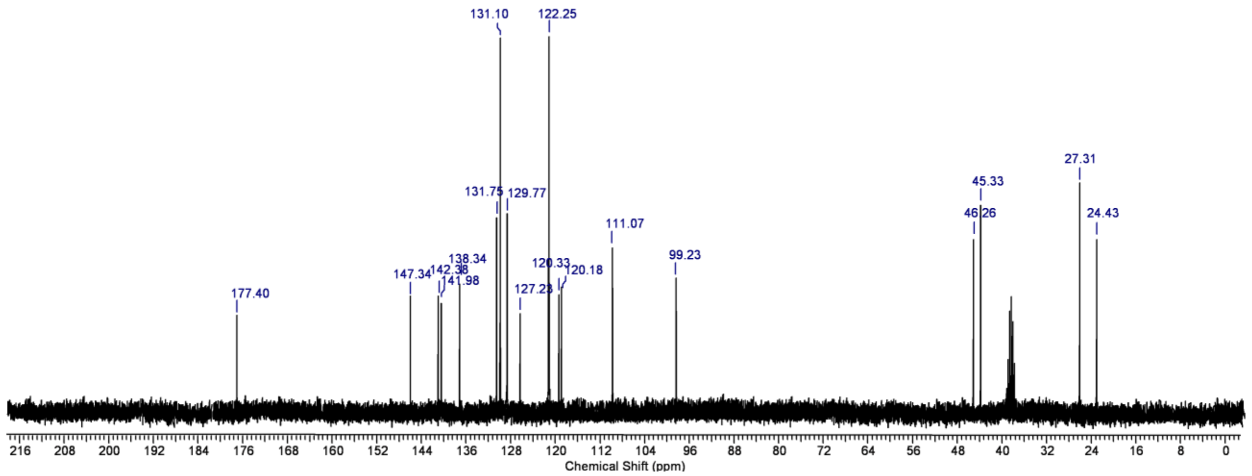

3-[5-(4-Bromophenyl)-2,3-dihydro-1*H*-pyrrolizin-6-yl]-1,3-dihydro-2*H*-indol-2-one (7c): brown powder, 17 %, mp 201-202 °C; ^{1}H NMR (200 MHz, DMSO-d_6) δ: 10.42 (s, 1H, 1-NH), 7.53 (d, *J*=8.6 Hz, 2H, 3,5-CH (4-bromobenzoyl)), 7.21 (d, *J*=8.6 Hz, 2H, 2,6-CH (4-bromobenzoyl), 6.97-6.70 (m, 5H, oxindol), 5.18 (s, 1H, 7-CH), 4.44 (s, 1H, 3-CH), 4.16-3.70 (m, 2H, 3-CH_2), 2.80-2.641 (m, 2H, 1-CH_2), 2.44-2.28 (m, 2H, 2-CH_2); ^{13}C NMR (75 MHz, DMSO-d_6) δ: 178.86 (2-CO), 141.81, 138.34, 135.49, 131.38, 129.47, 129.32, 127.24, 127.05, 124.30, 121.24, 120.21, 116.81, 109.69, 99.23, 46.26, 45.33, 27.31, 24.43. Anal. calcd. for $C_{21}H_{17}BrN_2O$ (393.28):C 64.13; H 4.36; N 7.12; Found: C 63.90; H 4.39; N 7.18.

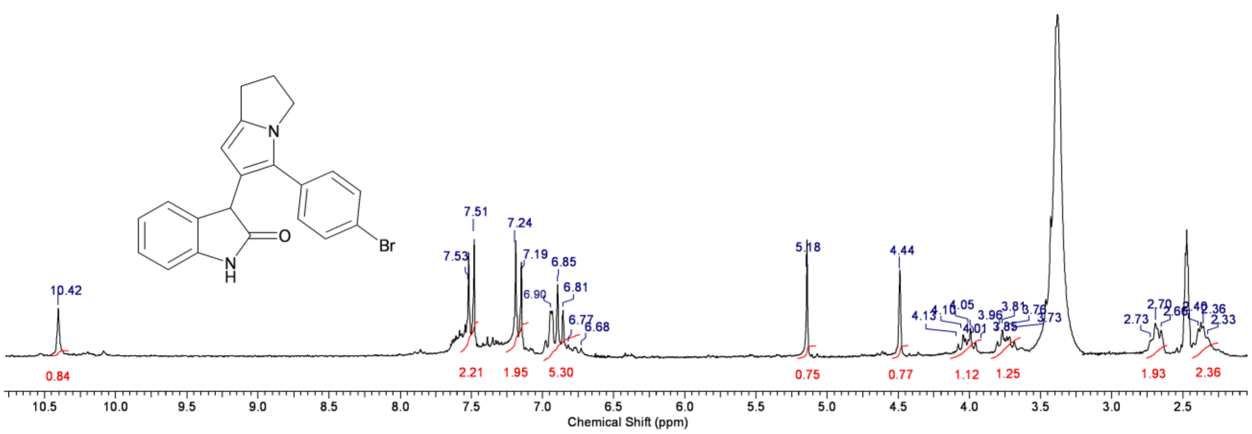
N
NH
O
Br
10.42
7.53
7.51
7.24
7.19
6.90
6.85
6.81
6.77
6.68
5.18
4.44
4.13
4.10
4.05
4.01
3.96
3.85
3.81
3.76
3.73
2.73
2.70
2.66
2.49
2.36
2.33
0.84
2.21
1.95
5.30
0.75
0.77
1.12
1.25
1.93
2.36
Chemical Shift (ppm)

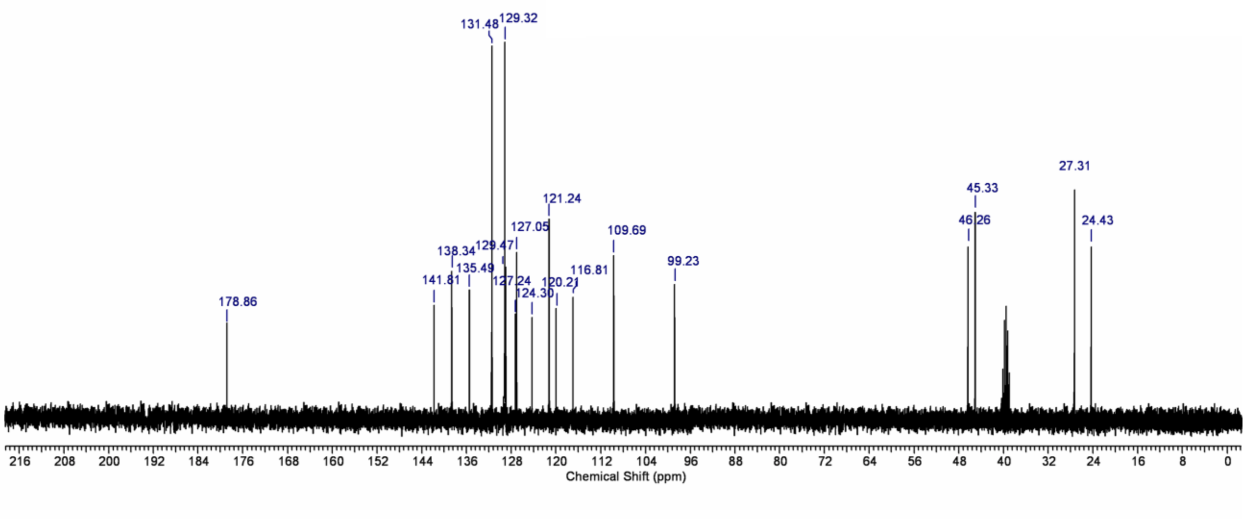
178.86
141.81
138.34
135.49
131.48
129.47
129.32
127.24
127.05
124.30
121.24
120.21
116.81
109.69
99.23
46.26
45.33
27.31
24.43
Chemical Shift (ppm)
