# Supplementary material for: The regioselective synthesis of spirooxindolo pyrrolidines and pyrrolizidines via three-component reactions of acrylamides and aroylacrylic acids with isatins and α-amino acids
Source: Beilstein J Org Chem. 2014 Jan 9;10:117–26. doi: 10.3762/bjoc.10.8 (PMC3896290; doi:10.3762/bjoc.10.8)
Supplement: File 2 — X-ray diffraction data description for compounds 4a and 6a. [file Beilstein_J_Org_Chem-10-117-s002.pdf]

Supporting information

for

The regioselective synthesis of spirooxindolo pyrrolidines and pyrrolizidines via three-component reactions of acrylamides and aroylacrylic acids with isatins and α-amino acids

Tatyana L. Pavlovskaya[1], Fedor G. Yaremenko[1,2], Victoria V. Lipson*[1,2,3], Svetlana V. Shishkina[1], Oleg V. Shishkin[1,3], Vladimir I. Musatov[1], Alexander S. Karpenko[4]

Address: [1]State Scientific Institution “Institute for Single Crystals” of National Academy of Sciences of Ukraine, 60, Lenin ave., Kharkov, 61178, Ukraine, [2]Antidiabetic Drug Laboratory, State Institution “V.J. Danilevsky Institute of Problems of Endocrine Pathology at the Academy of Medical Sciences of Ukraine”, 10, Artem St., Kharkov, 61002, Ukraine, [3]Organic Chemistry Department, V.N. Karazin Kharkov National University, 4, Svobody Sq., 61077, Kharkov, Ukraine, and [4]A.V. Bogatsky physico-chemical institute of the National Academy of Sciences of Ukraine, 86, Lustdorfskaya doroga, 65080, Odessa, Ukraine

Email: Victoria V. Lipson* - lipson@ukr.net

*Corresponding author

X-ray diffraction data description for compounds 4a and 6a

Molecular and crystal structure of the compound 4a.

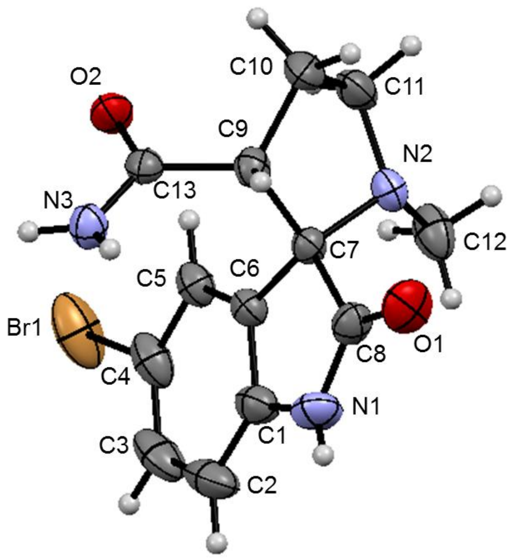

Figure S1: The molecular structure of the compound **4a** according to X-ray diffraction data. Thermal ellipsoids of atoms are shown at 50% probability levels.

The spiro-jointed dihydroindole and pyrrolidine rings are turned relatively to each other in such way that the angle between their mean planes is 98.6°. The pyrrolidine ring adopts an envelope conformation with the C11 atom deviated from the mean plane of the remaining atoms on −0.59 Å. The N2 atom has a pyramidal configuration, the sum of the bond angles centered at this atom is 338.3°. The carbamide substituent is located in equatorial position and is turned almost orthogonal to the plane of the pirrolidine ring (the N2–C7–C9–C13 and C7–C9–C13–O2 torsion angles are 127.2(2)° and −89.9(3)°, respectively). Such orientation of the carbamide substituent results in appearance of the C13...C5 intramolecular shortened contact (3.13 Å as compared with van der Waals radii sum [1] 3.42 Å)

In the crystal phase molecules **4a** are bonded by N–H..O, N–H…N and C–H…Br intermolecular hydrogen bonds:

N1–H…O2’ (x, 1+y, z) H…O 2.08 Å N–H…O 163°;

N3–H3Na…N2’ (1+x, y, z) H…N 2.26 Å N–H…N 163°;

N3–H3Nb…O1’ (1-x, 1-y, 2-z) H…O 2.28 Å N–H…O 161°;

C3–H…Br1’ (2-x, 1-y, 1-z) H…Br 3.05 Å C–H…Br 126°;

C5–H…Br1' (1-x, -y, 1-z) H…Br 3.01 Å C–H…Br 143°.

Molecular and crystal structure of the compound 6a.

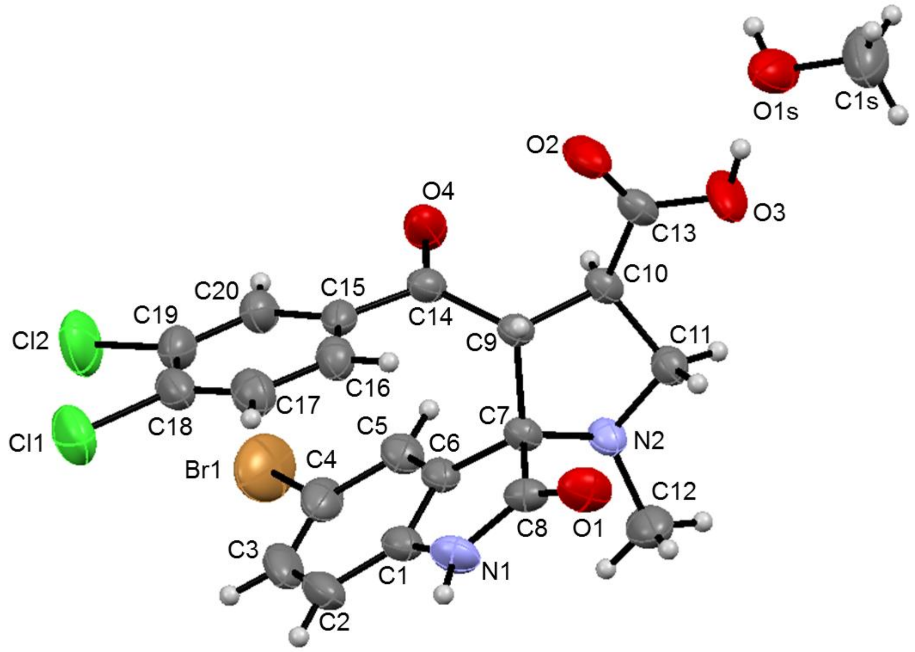

Figure S2: The molecular structure of the compound **6a** according to X-ray diffraction data. Thermal ellipsoids of atoms are shown at 50% probability levels.

The compound **6a** exists in the crystal phase as monosolvate with methanol. The spiro-jointed dihydroindole and pyrrolidine rings are turned relatively to each other in such way that the angle between their mean planes is 86.9°. The pyrrolidine ring adopts an envelope conformation with the C11 atom deviated from the mean plane of the remaining atoms on 0.61 Å. The N2 atom has a pyramidal configuration, the sum of the bond angles centered at this atom is 338.2°. The carboxylic substituent at the C10 atom is located in equatorial position and is slightly turned relatively to the C9–C10 endocyclic bond (the C7–C9–C10–C13 and C9–C10–C13–O2 torsion angles are −149.2(2)° and −32.1(4)°, respectively). The substituent at the C9 atom has equatorial orientation (the N2–C7–C9–C14 torsion angle is 122.7(2)°) and its carbonyl group is almost coplanar to the C9–C10 endocyclic bond (the C10–C9–C14–O4 torsion angle is 10.2(4)°). Such orientation of this substituent creates conditions for appearance of intramolecular stacking interaction between aromatic rings of indole fragment and substituent (the distance C6…C15 3.04 Å, C5…C14 3.14 Å and C1…C16 3.40 Å are shorter than the C…C van der

Waals radii sum 3.42 Å) leading to the disturbance of the conjugation between the carbonyl group and the aromatic ring in the substituent (the O4–C14–C15–C20 torsion angle is 25.9(4)°).

In the crystal phase molecules **16a** and solvate molecules of methanol are bonded by intermolecular hydrogen bonds O3–H…O1s' H…O 1.86 Å O–H…O 160°; N1–H…O2' (1+x, y, z) H…O 2.30 Å N–H…O 137°; O1s-H…O1' (x-1, y, z) H…O 2.08 Å O–H…O 164° and C1s-H1sb…Cl1' (x-1, y, z-1) H…Cl 2.68 Å C–H…Cl 139°.

Experimental part of X-ray diffraction study

The colourless crystals of **4a** ($C_{13}H_{14}N_3O_2Br$) are triclinic. At 293 K a = 6.9659(3), b = 7.5441(4), c = 13.4092(5) Å, α = 90.994(4)°, β = 90.947(4)°, γ = 106.218(5)°, V = 676.36(5) Å^3, M_r = 324.18, Z = 2, space group P $\bar{1}$, d_{calc}= 1.592 g/cm^3, μ(MoK$_\alpha$) = 3.040 mm^{-1}, F(000) = 328. Intensities of 6411 reflections (3942 independent, R_{int} = 0.018) were measured on an «Xcalibur-3» diffractometer (graphite monochromated MoK$_\alpha$ radiation, CCD detector, ω-scaning, 2Θ$_{max}$ = 60°).

The colourless crystals of **6a** ($C_{21}H_{19}N_2O_5BrCl_2$) are triclinic. At 293 K a = 8.8751(7), b = 10.5764(9), c = 12.352(1) Å, α = 75.858(5)°, β = 84.297(5)°, γ = 76.237(5)°, V = 1091.0(2) Å^3, M_r = 530.19, Z = 2, space group P $\bar{1}$, d_{calc}= 1.614 g/cm^3, μ(MoK$_\alpha$) = 2.165 mm^{-1}, F(000) = 536. Intensities of 15460 reflections (3842 independent, R_{int} = 0.043) were measured on an «Xcalibur-3» diffractometer (graphite monochromated MoK$_\alpha$ radiation, CCD detector, ω-scaning, 2Θ$_{max}$ = 50°).

The structures were solved by direct methods using the SHELXTL package [2]. The absorption correction was performed using the multi-scan method (T_{min} = 0.582, T_{max} = 0.751 for **4a** and T_{min} = 0.563 T_{max} = 0.671 for **6a**). Position of the hydrogen atoms were located from electron density difference maps and refined by "riding" model with U_{iso} = nU_{eq} of the carrier atom (n = 1.5 for methyl and hydroxy groups and n = 1.2 for other hydrogen atoms). Full-matrix least-squares refinement of the structures against F^2 in anisotropic approximation for non-hydrogen atoms using 3908 (**4a**), 3801 (**6a**) reflections was converged to: wR_2 = 0.111 (R_1 = 0.046 for 2795 reflections with F>4σ(F), S = 1.049) for structure **4a** and wR_2 = 0.110 (R_1 = 0.043 for 2435 reflections with F>4σ(F), S = 1.055) for structure **6a**. The final atomic coordinates, and crystallographic data for molecules **4a** and **6a** have been deposited at the Cambridge Crystallographic Data Centre, 12 Union Road, CB2 1EZ, UK (fax: +44-1223-

336033; e-mail: deposit@ccdc.cam.ac.uk) and are available on request quoting the deposition numbers CCDC 964021 for **4a** and CCDC 972494 for **6a**).

References

1. Zefirov, Yu. V. *Kristallographiya (Russian)*, **1997**, 42, 936–958.

2. Sheldrick, G. M., *Acta Crystallogr., Sect. A*, **2008,** *64*, 112–122. doi:10.1107/S0108767307043930
